# Giant Scintillation Yield Enhancement in Zero-Dimensional Halides by Exciton Confinement Manipulation

**DOI:** 10.34133/research.1230

**Published:** 2026-04-14

**Authors:** Yujie Wang, Xuemin Wen, Hongliang Shi, Eva Mihóková, Romana Kucerkova, Vladimir Babin, Jinlong Zhu, Jiawen Xiao, Martin Nikl, Xiaoping OuYang, Yuntao Wu

**Affiliations:** ^1^ State Key Laboratory of Functional Crystals and Devices, ShanghaiInstitute of Ceramics, Chinese Academy of Sciences, Shanghai 201899, China.; ^2^ School of Microelectronics, Shanghai University, Shanghai 201800, China.; ^3^ Center of Materials Science and Optoelectronics Engineering, University of Chinese Academy of Sciences, Beijing 100049, China.; ^4^ Department of Physics, Beihang University, Beijing 100191, China.; ^5^ FZU - Institute of Physics of the Czech Academy of Sciences, Prague 16200, Czech Republic.; ^6^ Beijing Key Lab of Microstructure and Property of Advanced Materials, College of Materials Science and Engineering, Beijing University of Technology, Beijing 100124, China.; ^7^ Northwest Institute of Nuclear Technology, Xi’an 710024, China.

## Abstract

Low-dimensional halides (LDHs) with self-trapped exciton (STE) emission are promising materials for scintillation applications. Nonetheless, for almost all LDHs, the measured scintillation yield is still far below theoretical value due to severe exciton–exciton/exciton–defect interaction under ionizing radiation, despite high photoluminescence quantum yield (PLQY). Here, we reported a substantial enhancement of scintillation yield in zero-dimensional (0D) Cs_3_YCl_6_ by structural modulation. By copper(I) alloying in Cs_3_YCl_6_, the delocalized excitons in [YCl_6_]^3−^ octahedra convert to strongly localized excitons within [Cu_2_(YCl_6_)_3_]^7−^ clusters in (Cs_8_Cu)Y_3_Cl_18_, as confirmed by first-principles calculations. Temperature-dependent photoluminescence spectroscopy and kinetic results reveal a higher energy barrier for STE quenching in (Cs_8_Cu)Y_3_Cl_18_ than in Cs_3_YCl_6_. Benefiting from the enhanced exciton confinement effect, (Cs_8_Cu)Y_3_Cl_18_ exhibits a 460% enhancement in the STE-related scintillation yield. This work opens up a new strategy to enhance scintillation yield in LDHs under ionizing radiation excitation.

## Introduction

Radiation detectors play an important role in diverse fields including homeland security, oil well logging, and medical imaging [1–4]. Scintillators, an integral part of radiation detectors, possess the capability to convert high-energy particles and rays into detectable low-energy ultraviolet–visible (UV-Vis) photons. Currently, methods to achieve bright scintillation are mainly oriented toward the externally activated mechanisms, represented by the commercialized NaI:Tl, LaBr_3_:Ce, and Lu_2_SiO_5_:Ce [5,6]. In these conventional scintillators, the charge carriers generated by ionization radiation exhibit strong delocalization within the lattice. It causes a high probability of interaction with defects, which in turn leads to low scintillation yield and high afterglow [7,8]. Thus, there is an urgent need for a material family capable of achieving strong localization of charge carriers under ionizing radiation [9,10]. Besides, these externally activated scintillation crystals, due to luminescent inhomogeneity caused by the segregation effect of dopant ions during crystal growth, will lead to an unavoidable issue of performance degradation in large-size crystals [11,12].

In recent years, a series of low-dimensional halides (LDHs), defined by the dimensionality of connectivity between luminescent polyhedra or units at the molecular level, have attracted growing attention. Luminescent LDHs have garnered significant attention due to their broad range of applications, particularly in x-ray detection and imaging [13–15]. These materials have become key subjects of research in materials science, energy, and chemistry, owing to their unique properties and potential for advanced technological applications [16–20]. These materials are of great interest due to their wide tunability of band gaps, excellent luminescence properties, high x-ray absorption coefficients, and good defect tolerance [21]. These advantages enable their wide applications in photovoltaics [22,23], light-emitting applications [24–26], and x-ray detectors [27,28]. These materials achieve efficient confinement of charge carriers through spatial restriction in isolated units, and the intrinsic self-trapped exciton (STE) emission from bromides or iodides combines to produce strong localized exciton emission [29–31]. Therefore, the LDHs with high radioluminescence (RL) efficiency from STEs are promising self-activated scintillators. In previous works, one-dimensional (1D) CsCu_2_I_3_ and Cs_5_Cu_3_Cl_6_I_2_ have exhibited a light yield of 16,000 photons/MeV and 64,800 photons/MeV, respectively [7,12]. Moreover, 0D Cs_3_Cu_2_I_5_ also achieves a light yield of 29,000 photons/MeV and an energy resolution of 3.4% at 662 keV [32]. Despite that a large amount of LDHs were developed, including Hf^4+^-/Zr^4+^-/Lu^3+^-/Y^3+^-/Sc^3+^-/La^3+^-/Sb^3+^-/Sn^2+^-/Cu^+^-/Ag^+^-based 0D/1D scintillators (Fig. S1), an important issue is overlooked. There is a significant gap between the practical scintillation yield and the theoretical value estimated by the Lempicki method [33]. This discrepancy in LDHs can be attributed to exciton–exciton and/or exciton–defect interactions under ionizing radiation [7,8,34,35]. Nonetheless, because of the lack of understanding the exciton manipulation mechanism, achieving scintillation yield enhancement in LDHs remains a grand challenge.

Many attempts to enhance scintillation yield of LDHs were made recently. For example, for 0D single crystals of Cs_3_Cu_2_I_5_, an exciton-harvesting strategy via externally Tl doping was proposed [8]. Due to the formation of strongly localized Tl-bound excitons, nonradiative quenching stemming from exciton–exciton interaction can be effectively suppressed, leading to a 300% increase of scintillation yield [8]. Nonetheless, doping strategy could introduce inhomogeneity of scintillation performance along the crystal growth direction due to segregation effect [7,12,36]. The ideal solution of enhancing scintillation yield in LDHs single crystals is by structural modulation strategy. It was found that different types of A-site cations, halogen anions, vacancy, and the building blocks could affect the exciton migration and intrinsic defects in 0D halides. It is known that mobile excitons can migrate easily via the Dexter resonant excitation energy transfer, resulting in trapping and nonradiative recombination at the intrinsic defects. In contrast, sufficient confined immobile excitons can prevent them from being dissociated at room temperature or even higher temperatures [29,34,37].

In this work, we propose an efficient strategy for enhancing scintillation yield in LDHs through the reinforcement of exciton confinement via structural modulation. Herein, we select 0D Cs_3_YCl_6_ with weak scintillation emission as a research target [38]. By partially replacing Cs^+^ in Cs_3_YCl_6_ with Cu^+^ to form (Cs_8_Cu)Y_3_Cl_18_, the luminescent units change from [YCl_6_]^3−^ units into paddle-wheel-like [Cu_2_(YCl_6_)_3_]^7−^ clusters. Detailed photo-physics studies and theoretical calculations of electronic structure and exciton dynamics clarify that the introduction of Cu facilitates exciton formation and regulation in 0D halides, resulting in a higher energy barrier for STE quenching. This modification results in a significant 460% increase in scintillation yield. It is associated with more localized excitons at the [Cu_2_(YCl_6_)_3_]^7−^ clusters. Finally, the prepared (Cs_8_Cu)Y_3_Cl_18_ @ polydimethylsiloxane (PDMS) scintillation film demonstrates superior x-ray imaging performance, particularly the high-temperature x-ray imaging capability. We believe that the exciton confinement enhancement strategy via structural modulation is an effective approach to tackle the strong exciton–exciton/defect issue in scintillation-oriented LDHs.

## Results and Discussion

### Structural modulation from Cs_3_YCl_6_ to (Cs_8_Cu)Y_3_Cl_18_

**Fig. 1.**
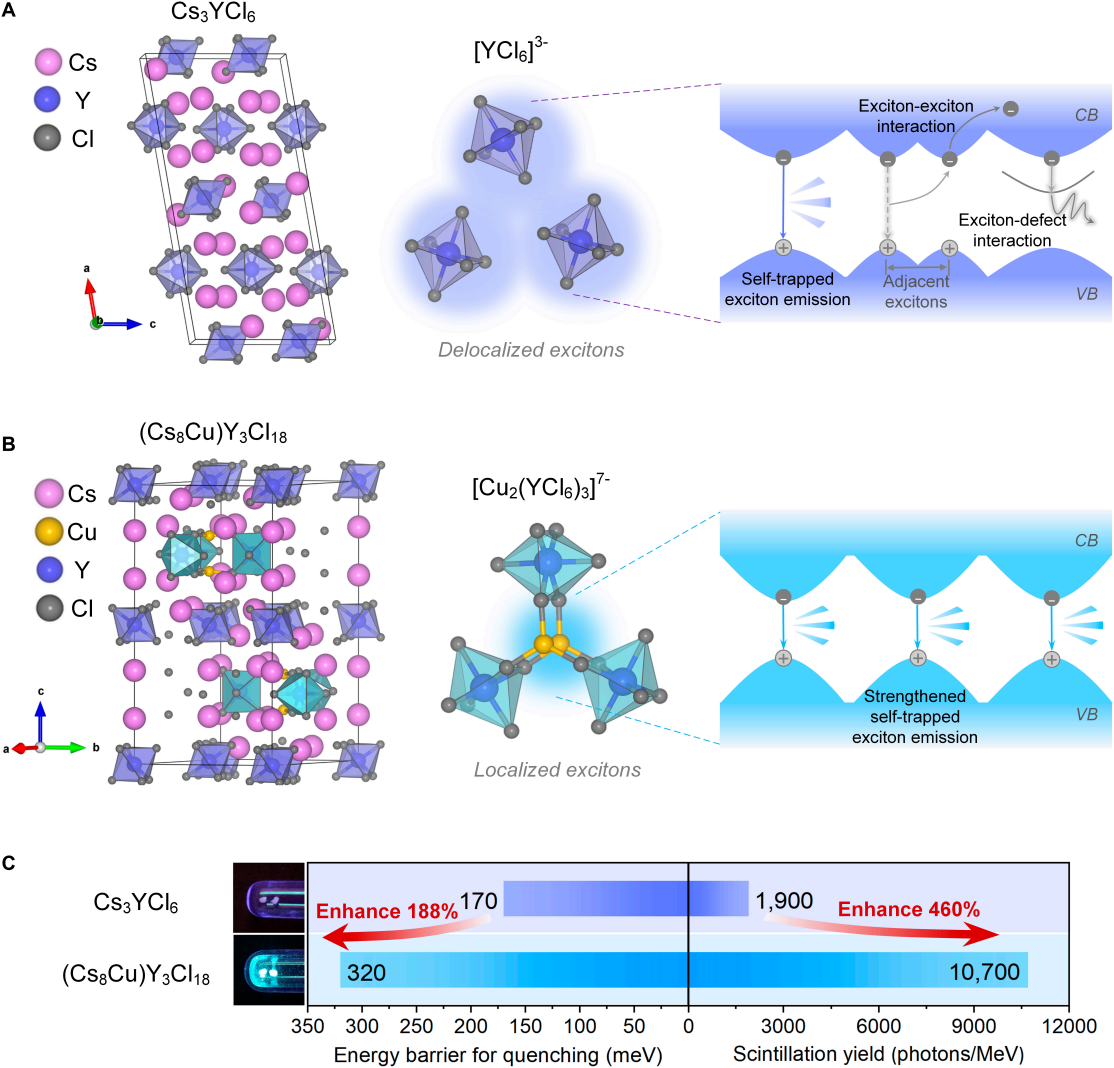
Exciton regulation via structural modification in low-dimensional halides. (A) Crystal structure of Cs_3_YCl_6_ (left), in which delocalized excitons are formed in isolated [YCl_6_]^3−^ octahedra (center). Under ionizing radiation, strong exciton–exciton interaction (EEI) and exciton–defect interaction (EDI) promote nonradiative recombination, resulting in weak emission (right). (B) Crystal structure of (Cs_8_Cu)Y_3_Cl_18_ (left), featuring [Cu_2_(YCl_6_)_3_]^7−^ clusters that confine excitons within a localized environment (middle). The reduced EEI/EDI and enhanced self-trapped exciton (STE) stabilization contribute to strong radiative recombination and efficient emission (right). (C) Single crystals of Cs_3_YCl_6_ and (Cs_8_Cu)Y_3_Cl_18_ under UV light (left), and comparison of energy barrier for quenching and STE-related scintillation yield between Cs_3_YCl_6_ and (Cs_8_Cu)Y_3_Cl_18_ (right).

The Cs_3_YCl_6_ and (Cs_8_Cu)Y_3_Cl_18_ single crystals (SCs) were obtained through the vertical Bridgman method. Details of the synthesis procedure and physical characterizations can be found in the Supplementary Materials (Figs. S2 to S4). The crystal structures of the Cs_3_YCl_6_ and (Cs_8_Cu)Y_3_Cl_18_ SCs were determined by the single-crystal x-ray diffraction (SCXRD) and are shown in the left part of Fig. 1A and B, respectively. For Cs_3_YCl_6_, each Y^3+^ is coordinated with 6 Cl^−^ ions, forming the [YCl_6_]^3−^ octahedron. These [YCl_6_]^3−^ octahedra are isolated from each other by Cs^+^ cations, resulting in a 0D structure at the molecular level (Fig. 1A). The structure of the unit changes when Cu is alloyed in the Cs_3_YCl_6_ system, resulting in a hexagonal crystal space group P/63m (*a* = 13.143 Å, *b* = 13.143 Å, *c* = 26.438 Å) for (Cs_8_Cu)Y_3_Cl_18_. It consists of 2 alternating layers. In one layer, isolated [YCl_6_]^3−^ octahedra are separated by Cs^+^ cations, similar to Cs_3_YCl_6_. In the other layer, 3 [YCl_6_]^3−^ octahedra connect with 2 Cu(I) ions to form a paddle-wheel-like [Cu_2_(YCl_6_)_3_]^7−^ cluster (Fig. 1B). These [Cu_2_(YCl_6_)_3_]^7−^ clusters are separated by Cs^+^ cations, ensuring that (Cs_8_Cu)Y_3_Cl_18_ forms a 0D structure. 0D halides with isolated units are prone to exhibit STE emission for the coupling of exciton with lattice vibrations upon excitation [10]. Since the spacing between the [Cu_2_(YCl_6_)_3_]^7−^ clusters in (Cs_8_Cu)Y_3_Cl_18_ is larger than that between the [YCl_6_]^3−^ octahedra in Cs_3_YCl_6_, clusters in (Cs_8_Cu)Y_3_Cl_18_ have smaller interactions with each other. Therefore, the excitons in (Cs_8_Cu)Y_3_Cl_18_ tend to be more localized than that in Cs_3_YCl_6_. Detailed crystallographic parameters and bond distances of Cs_3_YCl_6_ and (Cs_8_Cu)Y_3_Cl_18_ are summarized in Tables S1 and S2 for comparison. It is proven that the deviation between practical and theoretical light yield in LDHs is due to the presence of exciton–exciton interaction under high ionizing radiation [8]. In principle, the electronic coupling between [Cu_2_(YCl_6_)_3_]^7−^ clusters in (Cs_8_Cu)Y_3_Cl_18_ is expected to be much weaker than the [YCl_6_]^3−^ clusters in Cs_3_YCl_6_, which enables the suppression of exciton migration [29,39,40].

**Fig. 2.**
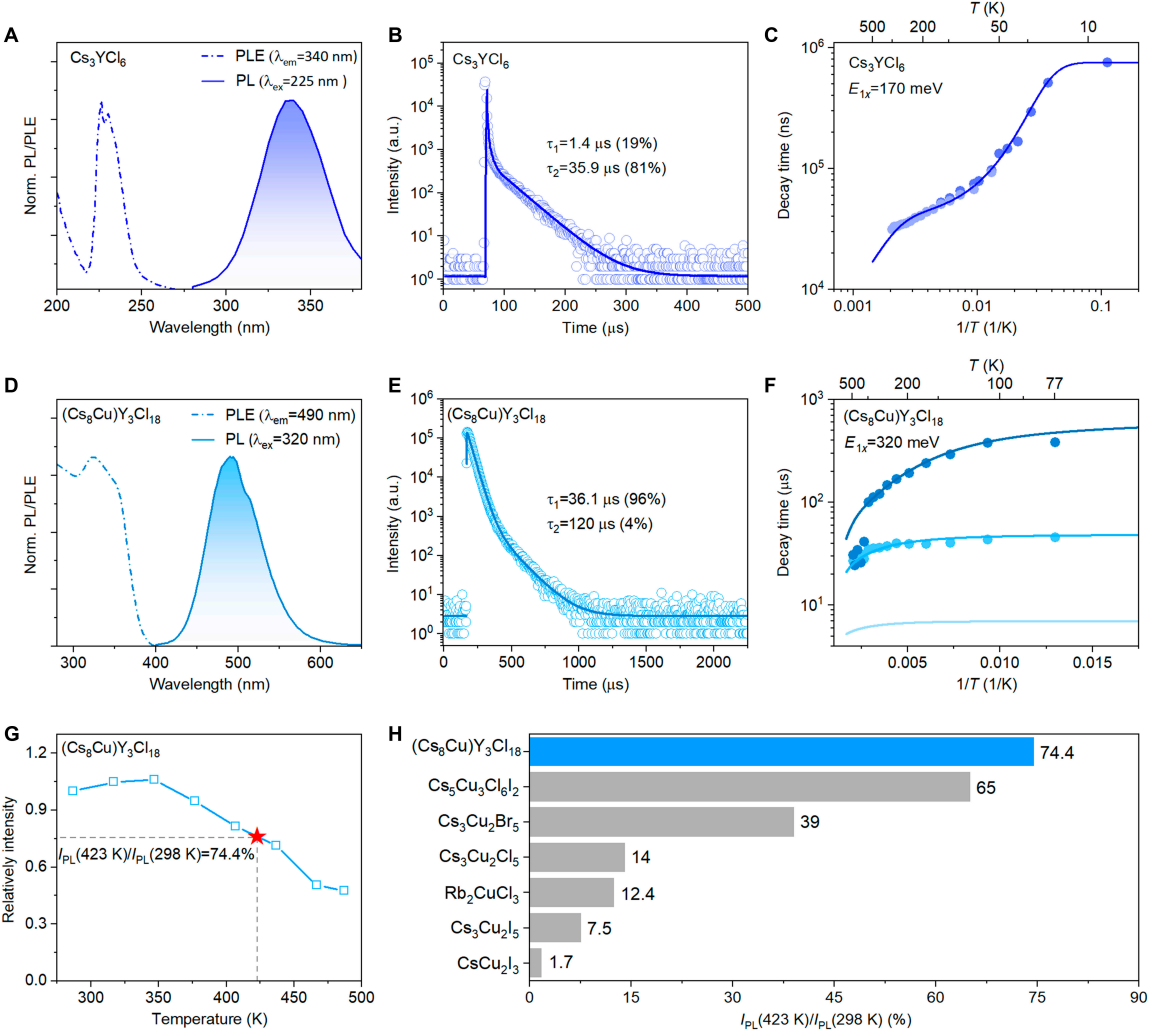
Photophysical properties of Cs_3_YCl_6_ and (Cs_8_Cu)Y_3_Cl_18_. (A) PL and PLE spectra of Cs_3_YCl_6_ at room temperature. (B) PL decay profile of Cs_3_YCl_6_ at room temperature. (C) Temperature dependence of PL decay times of 340-nm emission, under excitation at 200 nm, in Cs_3_YCl_6_. Solid circles are experimental data, and the solid line is the best fit of the 2-excited-level model in Fig. S9A to the data. The parameters used in the fit are as follows: *k*_1_ = 1,324 s^−1^; *k*_2_ = 5.7 × 10^4^ s^−1^; *K* = 2 × 10^5^ s^−1^; *D* = 10 meV. Quenching occurs from level 1 with the frequency factor *K*_1*x*_ = 1 × 10^6^ s^−1^ and energy barrier *E*_1*x*_ = 170 meV. (D) PL and PLE spectra of (Cs_8_Cu)Y_3_Cl_18_ at room temperature. (E) PL decay profile of (Cs_8_Cu)Y_3_Cl_18_ at 287 K. (F) Temperature dependence of PL decay times of center I (490 nm) in (Cs_8_Cu)Y_3_Cl_18_. Solid circles are experimental data, and solid lines are calculated decay components giving the best fit of the 3-excited-level model (as in [46]) in Fig. S11A to experimental data. Characteristic parameters of the model used for the calculated decay times are as follows: *k*_1_ = 1.5 × 10^3^ s^−1^; *k*_2_ = 2 × 10^4^ s^−1^; *k*_3_ = 1 × 10^5^ s ^−1^; *K* = 5 × 10^2^ s ^−1^; *D* = 4 meV; *K*_i_ = 2.2 × 10^4^ s ^−1^; *E* = 40 meV. Quenching occurs from level 1 with the frequency factor *K*_1*x*_ = 4 × 10^6^ s ^−1^ and energy barrier *E*_1*x*_ = 320 meV. For more details of the model, see the Supplementary Materials and references therein. (G) PL integral intensity of (Cs_8_Cu)Y_3_Cl_18_ as a function of temperature ranging from 287 K to 487 K. (H) Thermal stability of (Cs_8_Cu)Y_3_Cl_18_ compared with other copper-based halides [47–51].

As shown in Fig. 1C, 0D Cs_3_YCl_6_ crystals emit weak violet under UV light excitation. The energy barrier for quenching of Cs_3_YCl_6_ is 170 meV (Fig. 2C). In addition, the scintillation yield associated with STE was measured as 1,900 photons/MeV. In contrast, (Cs_8_Cu)Y_3_Cl_18_ emits bright cyan emission with a photoluminescence quantum yield (PLQY) of 86% under UV light excitation (Fig. S5). The energy barrier for quenching of (Cs_8_Cu)Y_3_Cl_18_ was enhanced and estimated to be 320 meV (Fig. 2F). Moreover, the RL intensity associated with STE in (Cs_8_Cu)Y_3_Cl_18_ increased nearly 460% compared to Cs_3_YCl_6_, reaching 10,700 photons/MeV (Fig. 1C). Although both Cs_3_YCl_6_ and (Cs_8_Cu)Y_3_Cl_18_ belong to 0D materials, their luminescent properties are different. The structural modulation achieved by Cu alloying may result in more localized electronic structure of [Cu_2_(YCl_6_)_3_]^7−^ clusters in (Cs_8_Cu)Y_3_Cl_18_.

### Photo-physics of Cs_3_YCl_6_ and (Cs_8_Cu)Y_3_Cl_18_

The photo-physics of Cs_3_YCl_6_ and (Cs_8_Cu)Y_3_Cl_18_ were then investigated. The photoluminescence emission (PL) and excitation (PLE) spectra of Cs_3_YCl_6_ and (Cs_8_Cu)Y_3_Cl_18_ are shown in Fig. 2A and D, respectively. Cs_3_YCl_6_ exhibits a broad STE emission at 340 nm, well seen also in RL spectra in Fig. S13, stemming from the 0D [YCl_6_]^3−^ octahedra isolated by Cs^+^ anions. PLE spectrum shows the band edge of Cs_3_YCl_6_ around 200 nm, which indicate Stokes shift larger than 2.5 eV. Well-separated excitation peak below the band edge around 230 nm yields similarly positioned luminescence around 340 nm, but with distinctly different temperature dependence of photoluminescence decays (compare Fig. 2C and Fig. S10C). For (Cs_8_Cu)Y_3_Cl_18_, at room temperature, only one emission center could be identified in the excitation and emission contour mapping (Fig. S6) along with the normalized PL and PLE spectra at room temperature (Fig. S7). (Cs_8_Cu)Y_3_Cl_18_ emits bright cyan emission, peaking at 490 nm (2.49 eV) with a full width at half maximum (FWHM) of 85 nm. Its Stokes shift is estimated as 1.3 eV from Fig. 2D, a typical feature for the STE emission, similar to other Cu-based LDHs [7,12,30–32,41,42]. Benefiting from its large Stokes shift and efficient cyan emission, (Cs_8_Cu)Y_3_Cl_18_ also shows potential for luminescence-related applications such as anti-counterfeiting and light-emitting diodes, as demonstrated by proof-of-concept experiments in the Supplementary Materials (Fig. S8). Temperature dependence of the dominant slow component decay time in the PL decay of Cs_3_YCl_6_ STE emission is shown in Fig. 2C. At room temperature (Fig. 2B), it can be approximated by 2 exponential fit with fast component of 1.4-μs decay time (19%) and a dominant slow component decay time of 35.9 μs (81%). For comparison, the PL decay of (Cs_8_Cu)Y_3_Cl_18_ monitoring upon 490-nm emission at 287 K shows well-resolved double exponential profile, which is kept through all the temperature period examined. It points to a complex energy level structure of STE. The decay of (Cs_8_Cu)Y_3_Cl_18_ yield a fast component of 36.1 μs (96%) and a slow component of 120 μs (4%) at 287 K (Fig. 2E). Such microsecond PL decay times further confirm that the 340-nm emission in Cs_3_YCl_6_ and the 490-nm emission in (Cs_8_Cu)Y_3_Cl_18_ both originate from triplet STEs [30]. Further support for the STE origin of 490-nm emission comes from the fact that the PL intensity of 490 nm exhibits linear dependence on the excitation power at room temperature, which rather rules out the possibility that the emission originates from a defect (Fig. S8) [30,41,43,44]. Therefore, as depicted in Fig. 1, the STEs in Cs_3_YCl_6_ and (Cs_8_Cu)Y_3_Cl_18_ are attributed to the isolated [YCl_6_]^3−^ octahedra and [Cu_2_(YCl_6_)_3_]^7−^ clusters, respectively. The Cu alloying may cause the excitons in the [Cu_2_(YCl_6_)_3_]^7−^ clusters to be more isolated than those in the [YCl_6_]^3−^ octahedra, thus exhibiting stronger emission.

Understanding the electron–phonon coupling in Cs_3_YCl_6_ and (Cs_8_Cu)Y_3_Cl_18_ is critical for interpreting their distinct luminescence properties. To investigate this, we studied the temperature dependence of PL spectra and decay profiles for both materials. In the case of Cs_3_YCl_6_ STE emission at 340 nm, to fit its temperature-dependent PL decay times of the dominant slow component, the 2-excited-level model (Fig. S10A) can provide satisfactory results with calculated energy barrier for STE quenching *E*_1*x*_ = 170 meV (Fig. 2C) [45].

For Cs_8_CuY_3_Cl_18_, the temperature dependences feature different behavior. PL emission at 77 K reveals the same main band at 490 nm (center I) and another weak band around 620 nm (center II). The excitation band edge of the former is long-wavelength shifted from 320 to 360 nm with increasing temperature (Fig. 2D and Fig. S11). At 77K, PLE spectrum of center II shows similarity with center I with low energy edge shifted to longer wavelength by 10 to 12 nm and no excitation beyond 380 nm. It quenches rapidly with temperature. Such PLE characteristics point to the defect-trapped exciton origin of 620-nm emission, and previous reports on Cu-based LDHs suggest the Cu-related defect [7]. The temperature-dependent PL decay time of (Cs_8_Cu)Y_3_Cl_18_ at 490 nm was summarized in Table S3. At 77 K, the PL decay of the 490-nm band shows 2 well-shaped components with decay times of about 45 μs (97.6%) and 380 μs (2.4%). The slower one gradually accelerates down to about 100 μs at 350 K, which points to 2 closely spaced excited level system with radiative lifetimes different by about one order. Emission intensity decreases and simultaneous acceleration of the slower component above 350 K without any sign of the delayed luminescence caused by the exciton ionization (thermal disintegration) point rather to thermal quenching from the lower level of the excited state and then to exciton disintegration, which means that exciton binding energy will be greater than thermal barrier for quenching. To fit both the temperature dependence of the PL intensity and the decay times of the 490-nm emission, we employed a 3-excited-level model (Fig. S11), which has been previously used for modeling Tl-bound excitons in CsCl:Tl, CsBr:Tl, and CsI:Tl [45,46]. In this model, the excited state consists of 2 closely spaced triplet levels (nos. 1 and 2) completed with the upper lying singlet level (no. 3) (Fig. S12A) [45]. Using such a model, successful fits of temperature dependences of both the decay times (Fig. 2F) and emission intensity (Fig. S12B) were obtained with parameters provided in the legend of these figures. The inclusion of singlet level appeared necessary to simultaneously fit temperature dependences of decay times and emission intensities, although we do not have direct experimental evidence of its existence from the decay curves themselves. It follows from the fits that the energy barrier for STE quenching is *E*_1*x*_ = 320 meV, which can be interpreted as either the binding energy of exciton *E*_b_ (quenching occurs due to exciton thermal disintegration) or energy barrier for thermal quenching from the excited to ground state of center I (Fig. 2F). In the latter case, the *E*_b_ value would be even higher. Besides, by fitting the temperature-dependent intensity, *E*_b_ is calculated to be 300 meV (Fig. S12B), which is similar to the result of fitting the decay time. These results indicate that (Cs_8_Cu)Y_3_Cl_18_ possesses a significantly higher energy barrier for STE quenching compared to Cs_3_YCl_6_, contributing to its superior thermal stability of luminescence. Even at 423 K, its PL intensity maintains almost 74.4% of that at 298 K (Fig. 2G). To the best of our knowledge, the luminescent thermal stability of (Cs_8_Cu)Y_3_Cl_18_ is the best among all copper-based halides ever reported, such as 65% for Cs_5_Cu_3_Cl_6_I_2_ [47], 14% for Cs_3_Cu_2_Cl_5_ [48], 12.4% for Rb_2_CuCl_3_ [49], 7.5% for Cs_3_Cu_2_I_5_ [50], and 1.7% for CsCu_2_I_3_ [51], as shown in Fig. 2H.

**Fig. 3.**
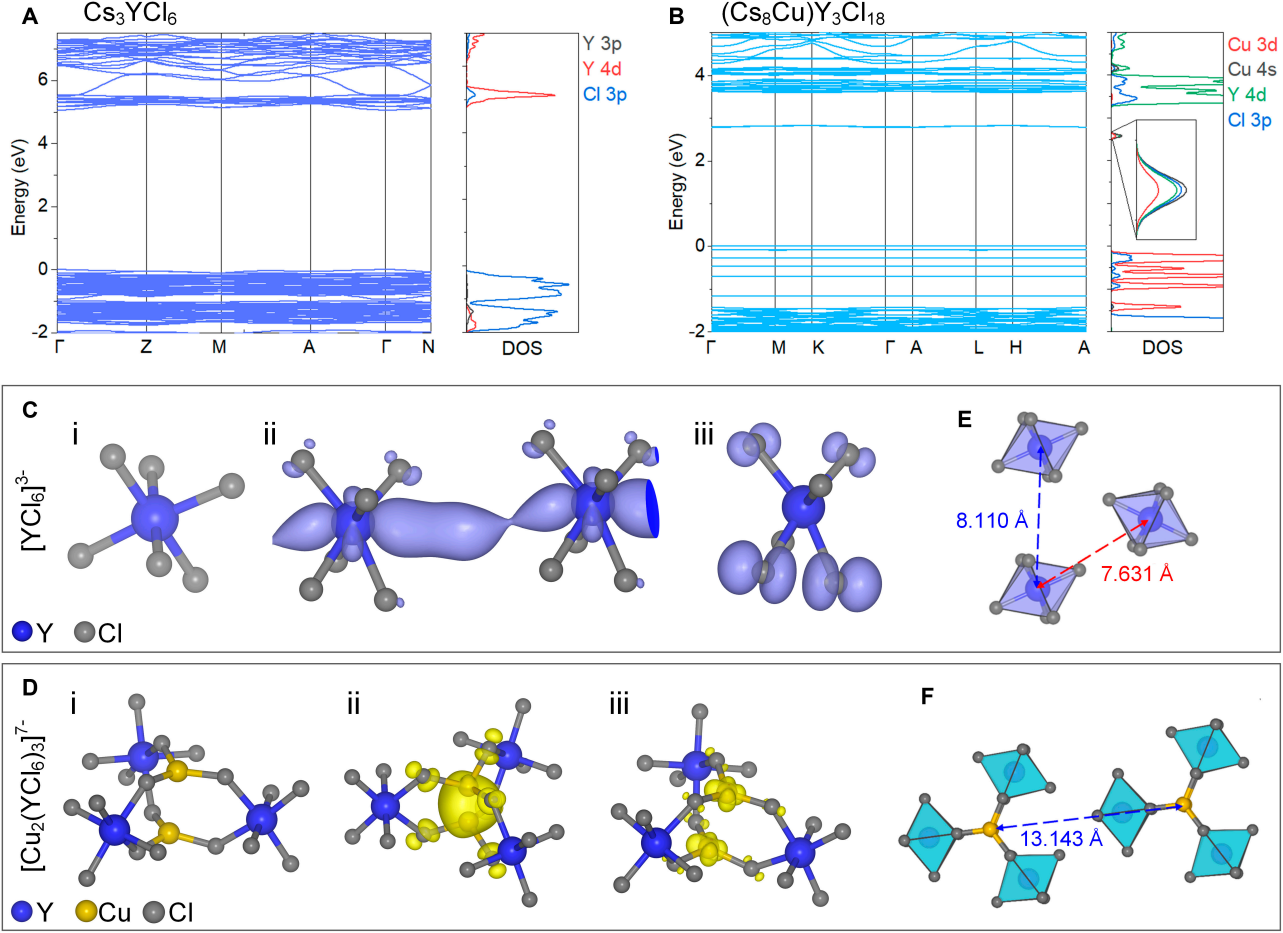
Calculations of electronic structure and exciton dynamics of Cs_3_YCl_6_ and (Cs_8_Cu)Y_3_Cl_18_. (A and B) Band structure and corresponding pDOS of Cs_3_YCl_6_ (A) and (Cs_8_Cu)Y_3_Cl_18_ (B). (C and D) Structure of the ground state (i), excited electron distribution (ii), and excited hole distribution (iii) of the [YCl_6_]^3−^ unit in (C) Cs_3_YCl_6_ and (D) [Cu_2_(YCl_6_)_3_]^7−^ clusters in (Cs_8_Cu)Y_3_Cl_18_ crystals. In (C), dark blue and gray spheres represent Y and Cl atoms, respectively. In (D), dark blue, gray, and yellow spheres represent Y, Cl, and Cu atoms, respectively. (E and F) Distance between adjacent units in [YCl_6_]^3−^ octahedra (E) and [Cu_2_(YCl_6_)_3_]^7−^ clusters (F), respectively.

We further elucidate the exciton localization effect from the theoretical calculation perspective. Detailed theory calculation procedure is illustrated in the experimental section of the Supplementary Materials. Our decomposed partial density of states (pDOS) shows that, for Cs_3_YCl_6_, the valence band mainly consists of Cl 3*p* states, while the conduction band is made of mainly Y 4*d* states and partially of Cl 3*p* states (Fig. 3A). For (Cs_8_Cu)Y_3_Cl_18_, the top of valence band mainly consists of Cu 3*d* and partially of Cl 3*p* states, while the lower edge of conduction band consists of Cu 4*s*, Cl *3p*, and Y *4d* orbitals, particularly the Cu 4*s* (Fig. 3B). Therefore, the electronic states of [YCl_6_]^3−^ units and [Cu_2_(YCl_6_)_3_]^7−^ clusters contribute mostly to the band edges of Cs_3_YCl_6_ and (Cs_8_Cu)Y_3_Cl_18_, respectively. Compared with Cs_3_YCl_6_, when the Cu ions are introduced into (Cs_8_Cu)Y_3_Cl_18_, due to the specific energies of atomic orbitals of Cu, the characters of band edge states are different in the above 2 compounds. Both the conduction band and valence band edges in (Cs_8_Cu)Y_3_Cl_18_ are dominated by the Cu ions (Fig. 3B). Due to isolation of [Cu_2_(YCl_6_)_3_]^7−^ cluster and [YCl_6_]^3−^ octahedron, the band structure of (Cs_8_Cu)Y_3_Cl_18_ and Cs_3_YCl_6_ is dispersion-less as shown in Fig. 3A and B, which favors the formation of localized excitons [45,48]. One significant difference of the band structure between (Cs_8_Cu)Y_3_Cl_18_ and Cs_3_YCl_6_ is the much narrower width of both the top of valence band and the bottom of conduction band of the former, i.e., narrow discrete bands, because of the much weaker coupling between the [Cu_2_(YCl_6_)_3_]^7−^ clusters than that of [YCl_6_]^3−^ octahedra in Cs_3_YCl_6_. The atomic structure of [Cu_2_(YCl_6_)_3_]^7−^ cluster in the ground state is shown in Fig. 3D (i). After excitation, the excited electrons mainly occupied the Cu 4*s* orbitals, which supplies an energy incentive to move 2 copper ions closer to enhance their hybridization (Fig. 3D, ii). Consequently, after the excitation of the electrons to the conduction bands, lattice distortion occurs within the [Cu_2_(YCl_6_)_3_]^7−^ cluster. Our calculations of structural relaxation further show that the 2 Cu ions move closer with the bond length of 2.34 Å compared with the perfect case of 3.32 Å, and correspondingly, 3 Cu–Cl bonds are elongated from 2.26 Å to 2.36 Å. For the STE in Cs_3_YCl_6_, 2 bonds of 6 Y-Cl in [YCl_6_]^3−^ cluster elongate about 0.22 Å, while the bond length of other 4 bonds changes negligibly (Fig. 3C).

As shown in Fig. 3D (ii), the excited electrons are mainly *s* orbital electrons of Cu ions and partial *p* orbital of Cl ions; the former mainly locates between the 2 Cu ions, which demonstrates the enhanced hybridization between them. The holes are mainly Cu *d* electrons, also very localized on the site of Cu ions (Fig. 3D, iii). Our calculated emission energy of STE is 2.27 eV, which is well consistent with our experimental result of 2.48 eV (center I). Our calculated binding energy of the STE (*E*_binding_ = *E*_STE_ − *E*_perfect cell_ − *E*_experimental gap_) is 0.31 eV, close to the experimental result of 0.29 eV. For Cs_3_YCl_6_, our calculated emission energies of STE are 3.14 eV (395 nm) and 3.08 eV (403 nm) for 2 nonequivalent Y sites and the former with a lower energy of 0.06 eV than the latter one. This is also close to the experimental emission peak at 3.64 eV (341 nm). As shown in Fig. 3C, the distribution of the excited electrons of STE in Cs_3_YCl_6_ is more spread than that of (Cs_8_Cu)Y_3_Cl_18_ (Fig. 3D), which indicates a much weaker interaction between the nearby STEs in the latter. Meanwhile, the distance between the nearest neighbor of [Cu_2_(YCl_6_)_3_]^7−^ clusters is much longer in (Cs_8_Cu)Y_3_Cl_18_ than that of [YCl_6_]^3−^ octahedra in Cs_3_YCl_6_, as shown in Fig. 3E and F (13.143 Å for [Cu_2_(YCl_6_)_3_]^7−^ clusters, and 8.110 Å or 7.631 Å for [YCl_6_]^3−^ octahedra). Therefore, compared with Cs_3_YCl_6_, the strongly localized STEs in (Cs_8_Cu)Y_3_Cl_18_ have much less probability to encounter nonradiative centers. Similarly, the semiconductors and insulators with narrow discrete band are also theoretically predicted to be potential high-light-yield scintillators in quaternary elpasolite compound like Cs_2_NaInBr_6_ with combination of large electronegativity difference among cations or anions and large nearest-neighbor distances in cation or anion sublattices [52,53].

### Scintillation properties and x-ray imaging applications of (Cs_8_Cu)Y_3_Cl_18_

**Fig. 4.**
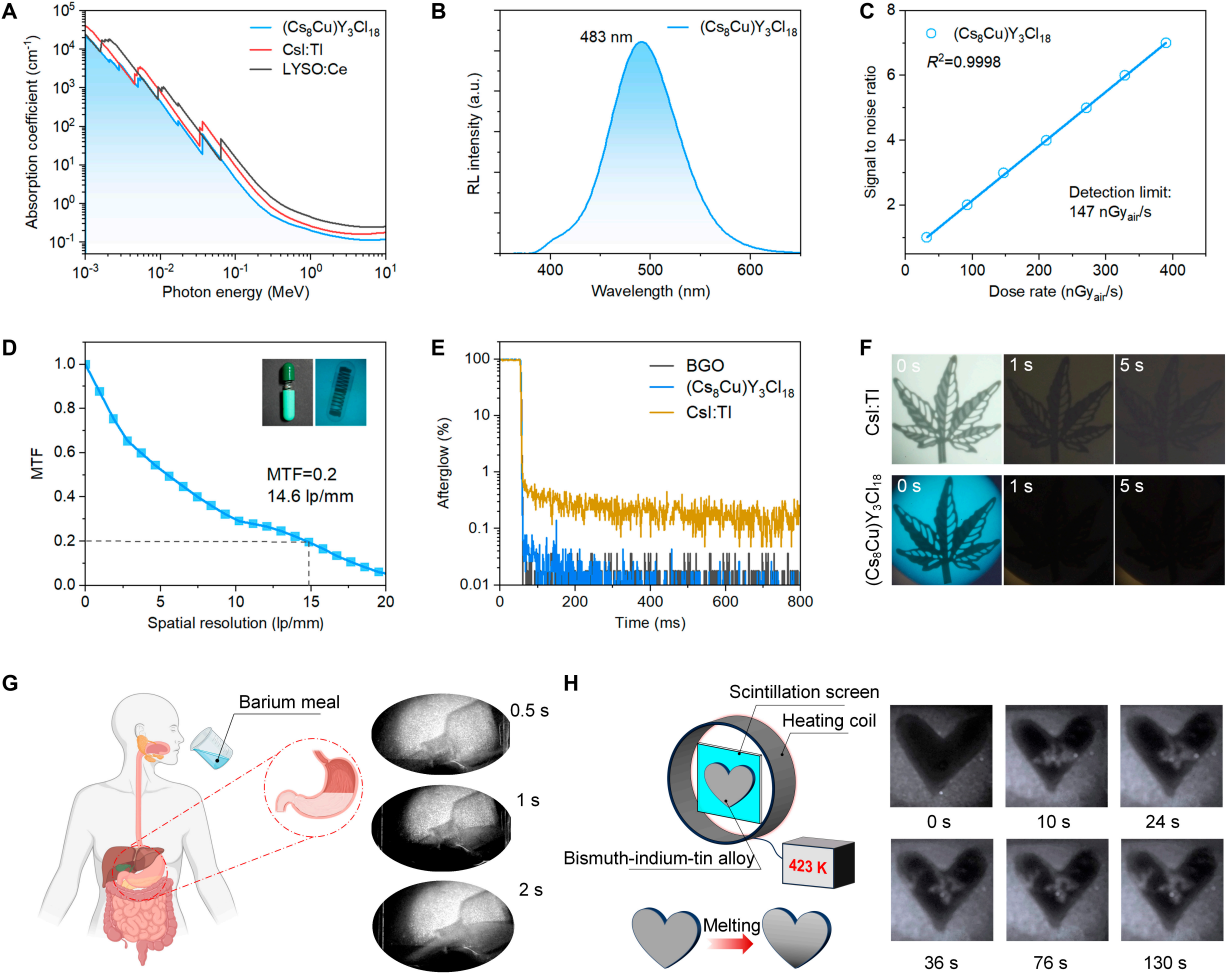
Scintillation properties and x-ray imaging performance based on (Cs_8_Cu)Y_3_Cl_18_ scintillation screen. (A) Absorption coefficients of (Cs_8_Cu)Y_3_Cl_18_ compared with CsI:Tl, LYSO:Ce, and CsPbBr_3_. (B) X-ray-excited RL spectrum of (Cs_8_Cu)Y_3_Cl_18_. (C) Response intensity as a function of dose rate for (Cs_8_Cu)Y_3_Cl_18_. (D) MTF of the x-ray images of the (Cs_8_Cu)Y_3_Cl_18_ scintillation screen. The inset shows spring in a capsule based on the (Cs_8_Cu)Y_3_Cl_18_ scintillation screen. (E) X-ray-induced afterglow profile of (Cs_8_Cu)Y_3_Cl_18_ compared with BGO and CsI:Tl benchmark scintillator. (F) Residual images of a metal flower using commercial CsI:Tl film and (Cs_8_Cu)Y_3_Cl_18_ film after 5 s of x-ray lasting excitation. (G) Schematic diagram of barium x-ray examination and the demonstration of barium x-ray examination of the gastrointestinal tract by using (Cs_8_Cu)Y_3_Cl_18_ scintillation film. (H) Schematic diagram of the high-temperature x-ray imaging apparatus and the melting process of the bismuth–indium–tin alloy at 480 K.

The scintillation application potentials of (Cs_8_Cu)Y_3_Cl_18_ were comprehensively examined. The absorption coefficients of (Cs_8_Cu)Y_3_Cl_18_ as a function of photon energies from 10^−3^ to 10 MeV were plotted in Fig. 4A by comparing with other classical scintillators. The absorption coefficient of (Cs_8_Cu)Y_3_Cl_18_ toward x-rays is comparable to that of 3D CsPbBr_3_ perovskite and commercialized inorganic scintillators, such as CsI:Tl and Lu_1.8_Y_0.2_SiO_5_:Ce. It indicates the potential application of (Cs_8_Cu)Y_3_Cl_18_ in the energy range for mammography (20 keV), radiography (20 to 50 keV), dental x-ray diagnostics (60 keV), and computer tomography (<100 keV) [32,54]. The x-ray excited RL spectrum exhibits an intense and broadband emission centered at 483 nm (Fig. 4B), similar to its PL spectrum in Fig. 2D. It suggests that there is no other radiative recombination channels under x-ray excitation for (Cs_8_Cu)Y_3_Cl_18_. The scintillation yield of (Cs_8_Cu)Y_3_Cl_18_ is estimated as 10,700 photons/MeV by comparing its RL spectrum integral with that of commercial Bi_4_Ge_3_O_12_ (BGO) reference measured under the same experimental conditions (the sample’s shape, reflection geometry, sufficient thickness about 1 mm to absorb all the energy from x-ray excitation). In the same way, the STE-related scintillation yield value of Cs_3_YCl_6_ was estimated as 1,900 photons/MeV (Figs. S13 and S14). Thus, a significant 460% enhancement of STE-associated scintillation yield is achieved in (Cs_8_Cu)Y_3_Cl_18_. As shown in Fig. S15, the absorption coefficients of Cs_3_YCl_6_ and (Cs_8_Cu)Y_3_Cl_18_ are similar. Consequently, the giant enhancement in (Cs_8_Cu)Y_3_Cl_18_ can be attributed to higher thermal stability of STEs facilitated by localized [Cu_2_(YCl_6_)_3_]^7−^ cluster compared with that in delocalized [YCl_6_]^3−^ octahedra.

The pulse x-ray excited scintillation decay profiles of (Cs_8_Cu)Y_3_Cl_18_ were measured and plotted in Fig. S16. The decay profiles can be well fitted by 2 components, 1.8 μs (24.7%) and 18.6 μs (75.3%). Due to the decent scintillation yield, (Cs_8_Cu)Y_3_Cl_18_ shows high signal-to-noise ratios (SNRs) under low-dose irradiation. As shown in Fig. 4C, the detection limit of (Cs_8_Cu)Y_3_Cl_18_ is estimated to be 147 nGy_air_ s^−1^. The value is 2 orders of magnitude lower than the x-ray medical diagnostics requirement (5.5 μGy_air_ s^−1^) [7]. The integral RL intensity remains unchanged compared with the initial level under cyclical x-ray irradiation at different tube voltages, indicating the superior radiation resistance of (Cs_8_Cu)Y_3_Cl_18_ (Fig. S17). We investigate the x-ray imaging performance based on uniform scintillation film by mixing the (Cs_8_Cu)Y_3_Cl_18_ powder with PDMS (Fig. S18). Through the x-ray phase-contrast imaging system illustrated in Fig. S19, a high spatial resolution of 14.6 lp/mm was obtained for (Cs_8_Cu)Y_3_Cl_18_ screen using a 1-mm-thick tungsten slide at a modulation transfer function (MTF) of 0.2 by the slanted-edge method (Fig. 4D and Fig. S20) [7,55–57]. The imaging performance was further visualized using a standard x-ray resolution pattern plate, with an observable resolution limit of approximately 14 to 16 lp/mm (Fig. S21A). The corresponding gray-value intensity change across the line pairs further confirms the excellent spatial resolution (Fig. S21B) [58,59], which is competitive with other reported metal halide PDMS composite films (Table S4) [41,60–62]. We also prepared the Cs_3_YCl_6_@PDMS composite scintillation film. However, due to the weak STE-related scintillation yield of Cs_3_YCl_6_, clear x-ray images could not be obtained under the same imaging conditions used for (Cs_8_Cu)Y_3_Cl_18_ (Fig. S22). Because of its high-resolution x-ray imaging performance, this (Cs_8_Cu)Y_3_Cl_18_@PDMS scintillation screen could reveal in great detail the metallic flower, microchip, and spring in capsule, thus possessing huge potential in nondestructive inspection and medical imaging (Fig. S23 and inset of Fig. 4D) [5,63]. The x-ray-induced afterglow profiles of (Cs_8_Cu)Y_3_Cl_18_ and commercial BGO and CsI:Tl scintillator are shown in Fig. 4E. (Cs_8_Cu)Y_3_Cl_18_ shows a comparable afterglow level with that of BGO throughout the test timescale (0 to 800 ms), which is far below CsI:Tl. Within the first 10 ms after x-ray cutoff, the residual afterglow signal of (Cs_8_Cu)Y_3_Cl_18_ is estimated to be 0.05% at 10 ms, which is nearly 2 orders of magnitude lower than that of widely used commercial CsI:Tl scintillators for x-ray imaging (~6% at 10 ms) [64,65]. Low afterglow intensity supports that scintillation yield is nearly equal to light yield because the emission under steady-state x-ray excitation (scintillation yield) includes both the fast component (light yield) and the slower/delayed component (afterglow) [4,64]. We further captured residual images of a metal flower by a digital camera after 5 s of x-ray lasting exposition (Fig. 4F). The (Cs_8_Cu)Y_3_Cl_18_ screen shows almost no residual images after x-ray cutoff, while the commercial Cs:Tl film still shows a clear image even after 5 s. Due to its negligible afterglow level, (Cs_8_Cu)Y_3_Cl_18_ could be used in dynamic x-ray imaging, which can avoid the ghosting effect resulting from afterglow [66].

Moreover, the applications of (Cs_8_Cu)Y_3_Cl_18_ scintillation screen in x-ray medical examination and high-temperature imaging were also studied. For x-ray medical examination application, we selected the barium x-ray, which is a radiographic examination of the upper gastrointestinal tract (Fig. 4G). In this examination, images of the outline of any part of digestive system, such as esophagus, stomach, and duodenum, could be produced through orally ingested barium meal contrast media when patients were exposed to x-ray radiation [67–69]. Due to the complexity of the human gastrointestinal system, here, as a conceptual experiment, we demonstrate the imaging process by instilling the barium meal contrast media into a disposable infusion set (Fig. S24). The barium meal liquid can be clearly shown up flowing into the bottom of the stomach, and the liquid level gradually rises to the pylorus and finally enters the duodenum. The contrast of the outline of the organs is significantly enhanced after the barium meal liquid coats the inside of the esophagus. Thus, if there is a tumor or gastric ulcer, it can be distinguished based on an irregular outline (Fig. 4G). It suggests the huge prospects of (Cs_8_Cu)Y_3_Cl_18_ scintillator in dynamic medical x-ray imaging when irradiated under a small amount dose. For the high-temperature imaging scenario, the imaging apparatus is illustrated in Fig. 4H. A bismuth–indium–tin alloy with a melting point of 423 K was used as the melting object at the temperature of 480 K (Fig. S25). The corresponding melting and morphological evolution of the alloy is recorded visually in Fig. 4H, which proves the x-ray imaging capability of (Cs_8_Cu)Y_3_Cl_18_ scintillator in harsh environments, such as detecting rock and fluid formations at high temperatures [70].

## Conclusion

In summary, we develop an exciton confinement strategy for scintillation-oriented LDHs by structural modulation. We demonstrate a giant enhancement of about 460% in STE-associated scintillation yield of (Cs_8_Cu)Y_3_Cl_18_ compared to that of 0D Cs_3_YCl_6_. The conformational and exciton localization changes that occur upon Cu alloying are unraveled. The formed [Cu₂(YCl_6_)₃]^7−^ clusters exhibit a more localized electronic structure than the [YCl_6_]^3−^ octahedra, resulting in a tighter binding of excitons within the clusters, thus shielding them from the influence of surrounding groups. Because of the enhanced scintillation yield with ultralow afterglow, and excellent thermal stability [*I*_PL_(423 K)/*I*_PL_(298 K) = 74.4%], the (Cs_8_Cu)Y_3_Cl_18_@PDMS composite scintillation film demonstrated promising applications in high-temperature x-ray imaging and medical diagnosis. More importantly, this work opens up a new horizon of designing efficient LDH scintillators with strong STE emission via structural modulation.

## Materials and Methods

### Synthesis of Cs_3_YCl_6_ and (Cs_8_Cu)Y_3_Cl_18_ crystals

Cesium chloride (CsCl; APL Engineered Materials, 99.99%), yttrium (III) chloride (YCl_3_; APL Engineered Materials, 99.99%), and copper(I) chloride (CuCl; Alfa Aesar, 99.99%) were used as received without further purification. Cs_3_YCl_6_ and (Cs_8_Cu)Y_3_Cl_18_ were prepared by the Bridgman method. The raw binary chlorides were weighed in stoichiometric ratio with a total mass of 3 g [CuCl in slight excess for (Cs_8_Cu)Y_3_Cl_18_] in the argon glovebox and sealed in a quartz ampoule. Then, the ampoules were sealed under a vacuum of 5 Pa and transferred into a Bridgman growth furnace. The ampoules were heated at 850 °C for 2 h and translated from the hot zone to the cool zone through a temperature gradient of approximately 40 °C/cm with a declining rate of 1 mm/h. After the growth completion, the furnace was cooled to room temperature with a rate of 10 °C/h. Note that because the crystals are sensitive to moisture, the whole handling process must be carried out in a dry environment.

### Fabrication of scintillation films

The (Cs_8_Cu)Y_3_Cl_18_ and Cs_3_YCl_6_ crystals were ground to powder by using agate mortar, and then the powder was filtered with 600 target standard sieves to get powder with a particle size of less than 23 μm, and the screened powder and PDMS were mixed in a mass ratio of 10:3. Then, the mixture was stirred on a magnetic mixer for 45 min so that the powder is fully dispersed in the PDMS. Finally, the mixture gel was placed in a glass plate and heated at 90 °C for 9 h on the heating panel. Note that the whole process must be carried out in the argon glovebox.

### Fabrication of the heart-shaped metal alloy

The heart-shaped metal alloy used in Fig. 4H was fabricated from a low-melting-point bismuth–indium–tin alloy with a melting point of approximately 423 K. The alloy was heated using a ring-shaped heating mantle (custom-built heater, Zhengzhou Yifeng Electric Heating Element Co. Ltd.) until fully melted and subsequently poured into a custom-made graphite mold for casting and cooling. The graphite mold was prepared by manually carving a heart-shaped cavity with a depth of ~3 mm into a flat graphite block. After solidification, the obtained alloy was mechanically polished with sandpaper and thinned to achieve a smooth surface finish.

### Reference scintillators

The commercial BGO and CsI:Tl scintillators used for comparison in this work were supplied by the R&D Center of the Shanghai Institute of Ceramics, Chinese Academy of Sciences.

### Material characterization

Single-crystal x-ray diffraction (SCXRD) data were measured on a Bruker–Lynxeye diffractometer with a graphite-monochromatized Mo K𝛼 radiation (𝜆 = 0.71 073 Å) at 100 K. Powder x-ray diffraction (PXRD) was carried out on a D8 ADVANCE x-ray diffractometer using Cu Kα radiation (λ = 1.5418 Å). The diffraction data were collected over a 2θ range of 10° to 70° with a step size of 0.02° and a scan rate of 5° min^−1^. X-ray photoelectron spectroscopy (XPS) measurement was performed on a Thermo Scientific K-Alpha with an x-ray source of Al Kα (hν = 1,486.6 eV). Energy-dispersive spectrometer (EDS) mapping was conducted by a Verios G4 high-resolution field-emission scanning electron microscope. The diffuse reflection spectra were recorded by a UV–Vis–NIR (near-infrared) spectrometer over the range from 200 to 800 nm (Hitachi High-Tech Science Corporation, UH4150), and BaSO_4_ powder was used as a reference.

The photoluminescence excitation (PLE) and emission (PL) contour mapping and spectra were measured with a Horiba FluoroMax+ spectrofluorometer. Temperature dependence of photoluminescence decay curves and spectra were measured at the Horiba Jobin Yvon 5000M spectrofluorometer using xenon steady-state and microsecond pulsed xenon lamps. All the measured spectra are recalculated using appropriate calibration curves of the excitation/emission setup parts to remove experimental distortions. The excitation power-dependent PL spectra were obtained by using an Edinburgh FLS1000 fluorescence spectrophotometer combined with an LE-LS 304 type 304-nm laser (Shenzhen LEO-Photoelectric Co. Ltd.) as the excitation source. PLQY was measured using a QE-2100 system (Otsuka Electronics, Japan) equipped with an integrating sphere. Approximately 0.3 to 0.5 g of sample powder were pressed into a custom sample holder with a diameter of 1.25 cm and a depth of 2 mm. First, the baseline spectrum was recorded using BaSO_4_ as a reference. Then, the sample spectrum was collected under identical conditions. The integrated area of the excitation light for BaSO_4_ is labeled as S_1_ and that for the sample is labeled as S_2_, and the integrated area of the emitted luminescence of the sample is labeled as S3. PLQY was calculated using the following formula: PLQY = S_3_/(S_1_ − S_2_). Further details of this measurement procedure can be found in our previous work [71].

### Scintillation characterization

The x-ray excitation RL spectra were measured with a tungsten anode x-ray tube (40 kV, 15 mA) as the excitation source and an Ocean Optical charge-coupled device spectrometer as the detector. Similarly to PL ones above, the measured spectra are recalculated using embedded calibration curves to remove experimental distortions. The afterglow profiles and radiation stability measurement were measured by the combination of a Horiba FluoroMax+ spectrofluorometer and the abovementioned x-ray tube excitation source. Scintillation decays were recorded under the pulse x-ray excitation. The detection limit was evaluated utilizing a silicon-based optical power meter model 843-R (Newport), while rays from the portable x-ray source (tungsten anode target, MOXTEK, TUB00153-9) were attenuated by copper foil.

### X-ray imaging

In x-ray scintillation imaging, images were reflected by a prism before being captured by a commercial digital camera (SONY, ILCE-7RM2) combined with a MACRO lens (SONY, SEL50M28). The spatial resolution of the x-ray imaging system was estimated based on a standard x-ray line pair card (0.03mmPb, CN89729, made in Germany).

### Theoretical calculations

Our first-principles electronic structure calculations are performed by using the Heyd–Scuseria–Ernzerhof (HSE) hybrid functional implemented in the Vienna ab initio simulation package (VASP) [72,73]. The HSE functional mixes semilocal exchange with a fraction of nonlocal Hartree–Fock exchange, in which the exchange interaction is further separated into short-range and long-range components by a screening parameter. This treatment effectively reduces the self-interaction error and is known to significantly improve the description of electronic structures and band gaps in semiconductors and insulators. The fraction for the nonlocal Hartree–Fock exchange is set to be the default value of 0.25. The total energy of an exciton was calculated by fixing the occupation numbers of the electron and hole-occupied eigenlevels [∆ self-consistent field (∆SCF) method] [74,75]. This approach allows a direct evaluation of exciton-related total energies within the same theoretical framework as the ground-state calculations. Following the Franck–Condon principle, the exciton excitation and emission energies were obtained by calculating the total energy differences between the excited and the ground states using HSE-optimized ground-state and excited-state structures, respectively. The cutoff energy for the plane wave basis was set at 300 eV, and the atomic positions were fully relaxed until the residual forces are less than 0.02 eV/Å. The 120- and 80-atom unit cell with experimental lattices (*a* = *b* = 13.143 Å and *c* = 26.438 Å) and (*a* = 26.7 Å, *b* = 8.110 Å, and *c* = 12.929 Å) is used for (Cs_8_Cu)Y_3_Cl_18_ and Cs_3_YCl_6_, respectively. Brillouin zone (BZ) integrations are performed using a Γ-point sampling scheme, which is sufficient given the large unit-cell sizes of the studied systems.

## Data Availability

Data used to support the findings of this study are included within the article and Supplementary Materials files.

## Supplementary Materials

Supplementary 1 Experimental details Tables S1 to S4 Figs. S1 to S25
