# Supplementary material for: Giant Scintillation Yield Enhancement in Zero-Dimensional Halides by Exciton Confinement Manipulation
Source: Research (Wash D C). 2026 Apr 14;9:1230. doi: 10.34133/research.1230 (PMC13077130; doi:10.34133/research.1230)
Supplement: Supplementary 1 — Experimental details Tables S1 to S4 Figs. S1 to S25 [file research.1230.f1.zip › Research_SI_clean_20260217.docx]

**Supplementary materials**

**Experimental details**

**Tables S1 to S4**

**Figs. S1 to S25**

**Difference between theoretical and measured light yield in 0D/1D halide scintillators**

The yields under X-/γ- ray irradiation and PLQYs of 0D/1D halide scintillators are summarized according to the reported literatures. The theoretical light yields are calculated according to the band gap value using the Bartram-Lempicki model as described in our previous work^1^:

$$\begin{aligned} LR=\frac{S*Q}{\beta*E_{g}}\ldots\ldots\ldots\ldots\ldots\ldots\ldots\ldots\ldots\ldots\ldots\ldots\ldots\ldots..1 \end{aligned}$$

where *S* and *Q* represent transport/transfer efficiency of the e–h pair energy to the emission centers and the internal quantum efficiency in emission centers, respectively. Here, the values are considered as 1. *β* is 1.5–1.8 for ionic halide compounds, and the approximate mean is used as 1.65.


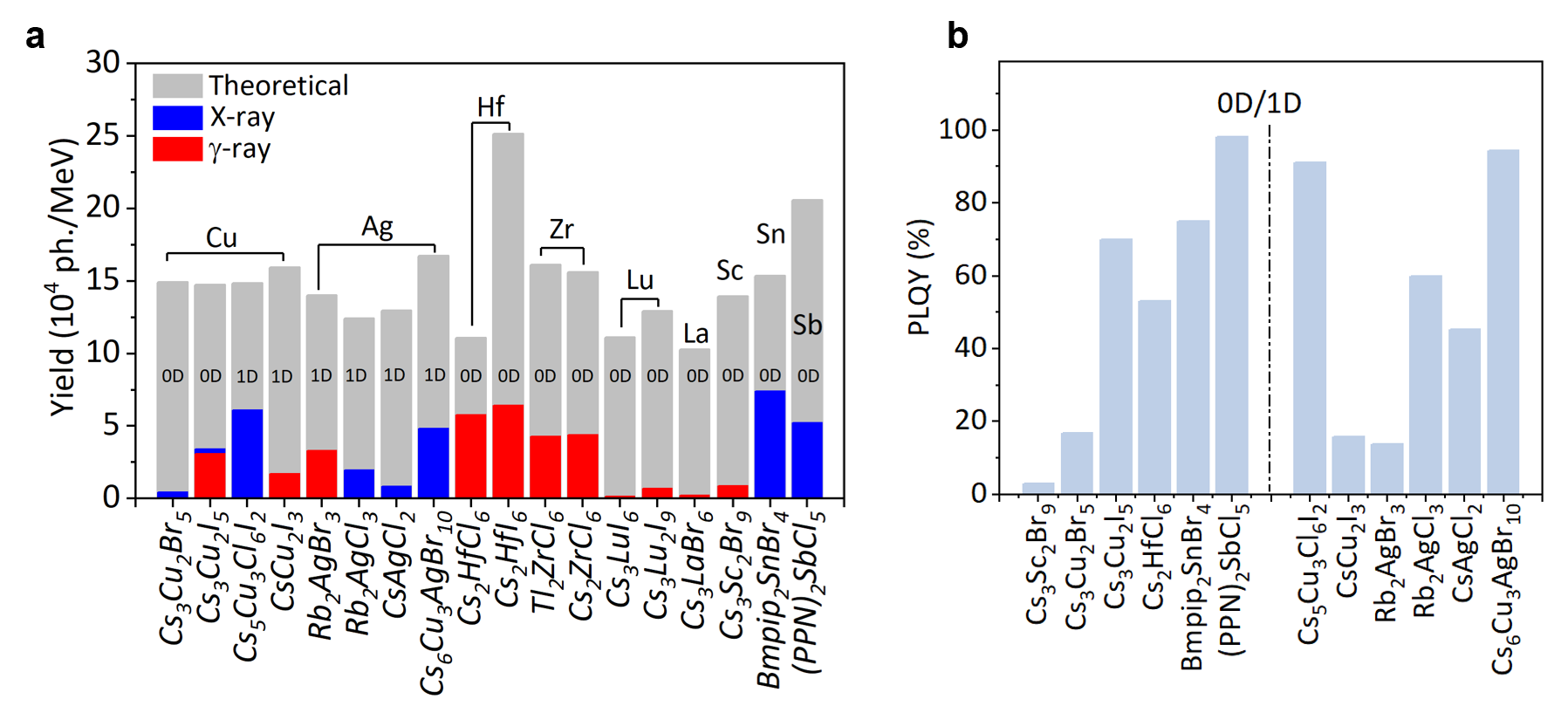


Figure S1. a, Yields of LDH scintillators.^1, 2, 3, 4, 5, 6, 7, 8, 9, 10, 11, 12, 13, 14, 15, 16, 17, 18, 19, 20, 21, 22, 23, 24, 25^ b, PLQYs of LDH scintillators.^1, 2, 3, 4, 5, 6, 7, 8, 9, 10, 11, 12, 13, 14, 15, 16, 17, 18, 19, 20, 21, 22, 23, 24, 25^

**Characterization of composition and chemical nature of Cs_3_YCl_6_ and (Cs_8_Cu)Y_3_Cl_18_ single crystals.**

The phase purity of Cs_3_YCl_6_ and (Cs_8_Cu)Y_3_Cl_18_ was investigated by the powder X-ray diffraction (PXRD) (**Figure S2**). PXRD patterns of Cs_3_YCl_6_ and (Cs_8_Cu)Y_3_Cl_18_ match well with the simulation results from SCXRD with no visible diffraction peaks from the second phase or purities. Furthermore, quantitative energy dispersive spectroscopy (EDS) and elemental mapping characterizations (**Figure S3**), combined with X-ray photoelectron spectroscopy (XPS) results (**Figure S4**) confirm the chemical composition and valence state in (Cs_8_Cu)Y_3_Cl_18_.


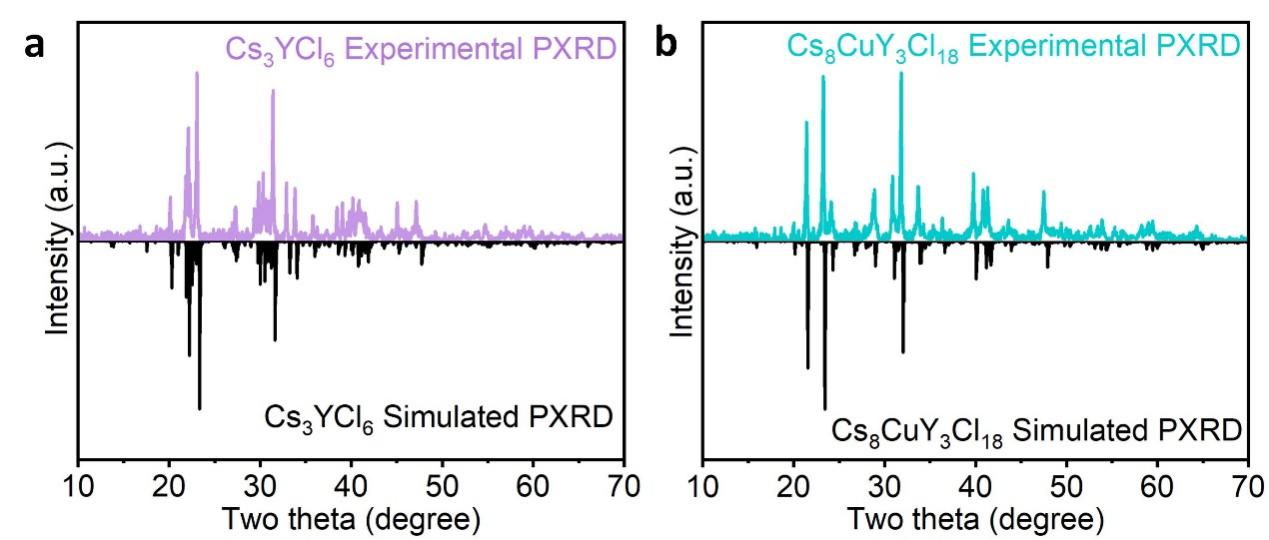


Figure S2. a, PXRD patterns and simulated patterns from SCXRD of Cs_3_YCl_6_ single crystals. b, PXRD patterns and simulated patterns from SCXRD of (Cs_8_Cu)Y_3_Cl_18_ single crystals.


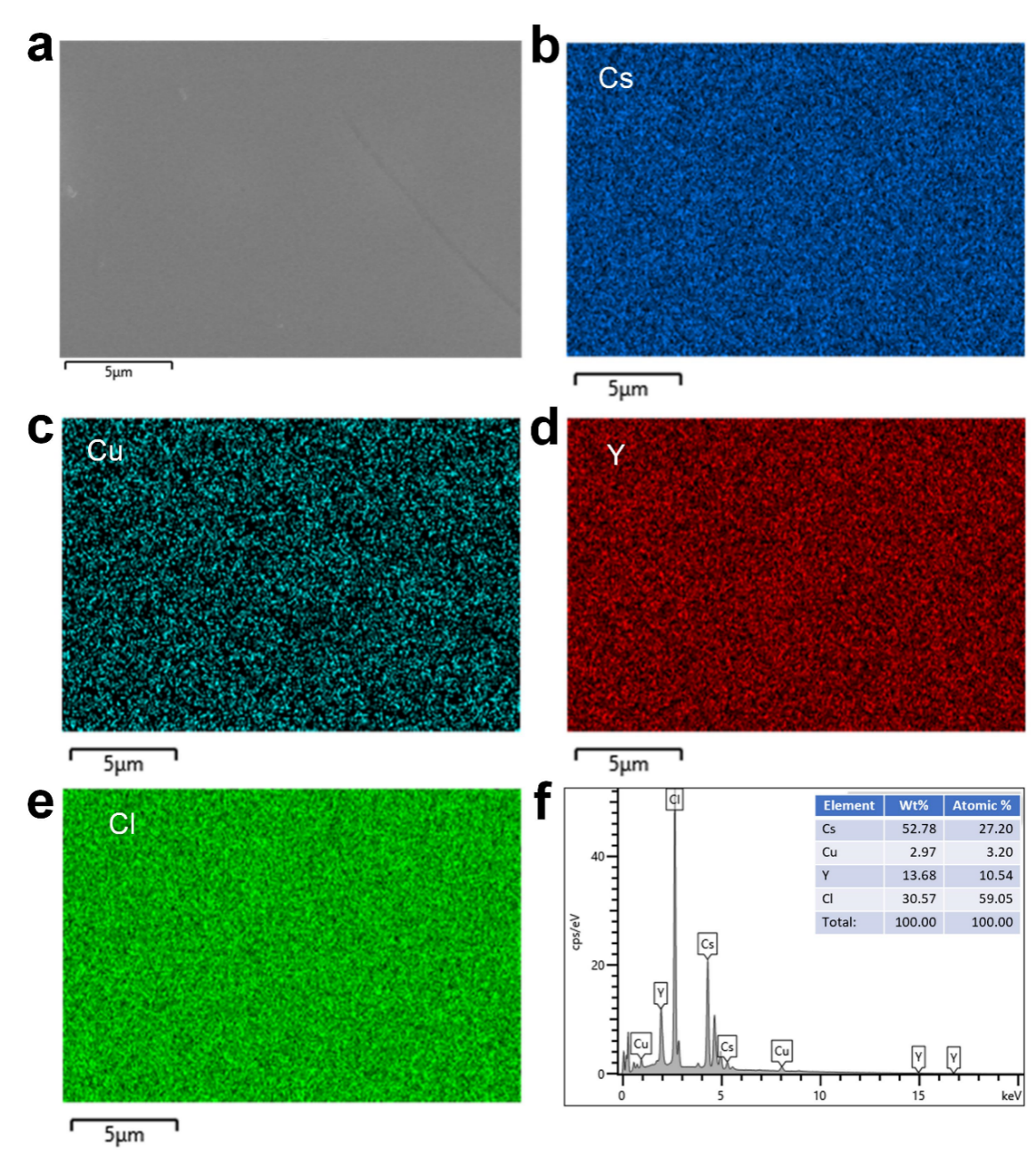


Figure S3. a-e, Elemental mapping images of (Cs_8_Cu)Y_3_Cl_18_ crystal. f, Energy dispersive spectrum (EDS) of (Cs_8_Cu)Y_3_Cl_18_ and the percentage of element content (inset table).

The elemental mapping images in **Figure S3 a-e** reveal the homogeneous element distribution of Cs, Cu, Y, and Cl in (Cs_8_Cu)Y_3_Cl_18_, ruling out the existence of other phases. The EDS characterization in **Figure S3 f** confirms that the atomic ratio of Cs, Cu, Y, and Cl is 8.5:1:3.3:18.5, which is in good agreement with the stoichiometry in (Cs_8_Cu)Y_3_Cl_18_.


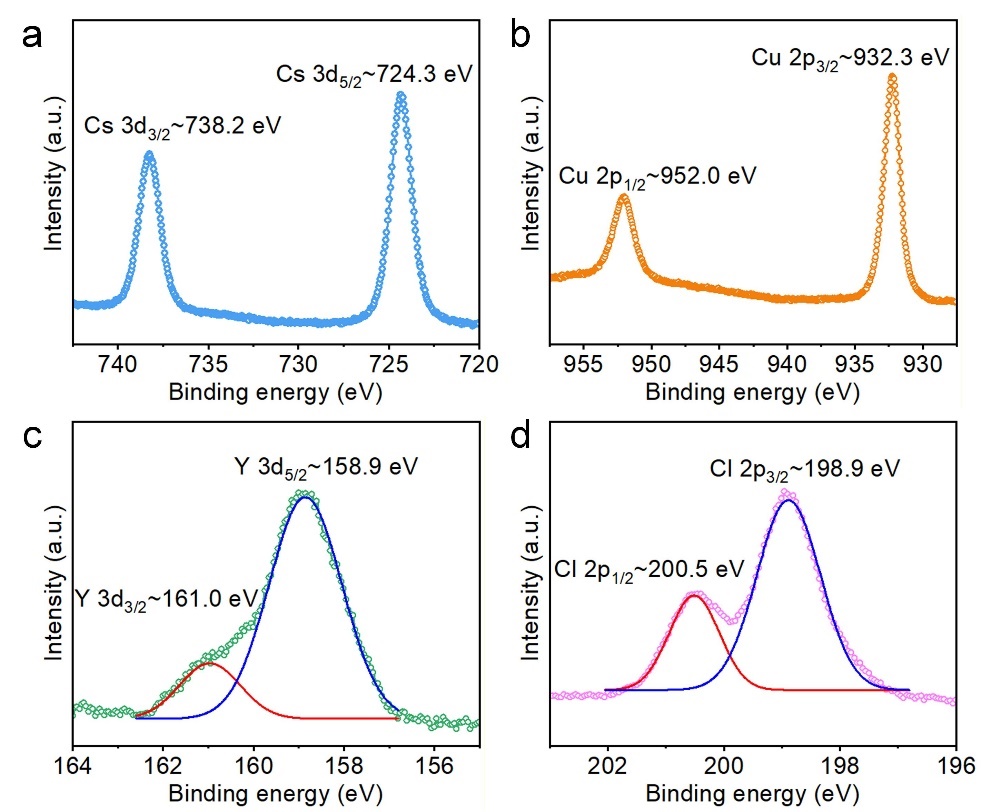


Figure S4. The X-ray photoelectron spectrum (XPS) of (Cs_8_Cu)Y_3_Cl_18_. Magnified XPS spectra of Cs (a), Cu (b), Y (c) and Cl (d), respectively.

All four elements are in the expected valence states. Particularly, the Cu 2p region exhibits two characteristic peaks with binding energies of 952.0 eV (2p_1/2_) and 932.3 eV (2p_3/2_), consistent with the nature of Cu^+^.^26^ Two characteristic peaks of Y 3d are 161 eV (3d_1/2_) and 158.9 eV (3d_5/2_), agreeing well with Y^3+^. Additionally, the binding energy of Cs 3d (738.2 eV for Cs 3d_3/2_, 724.3 eV for Cs 3d_5/2_) and Cl 2p (200.5 eV for Cl 2P_1/2_, 198.9 eV for Cl 2P_3/2_) are consistent with Cs^+^ and Cl^-^, respectively.^9^


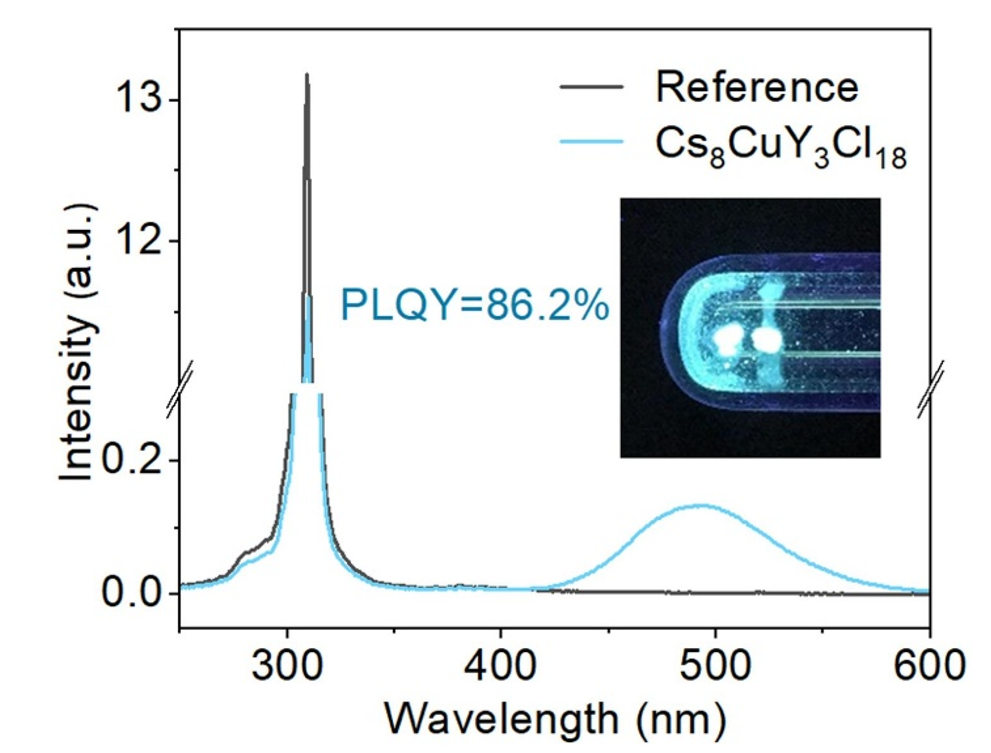


Figure S5. The PLQY spectra of (Cs_8_Cu)Y_3_Cl_18_ under 310 nm excitation.


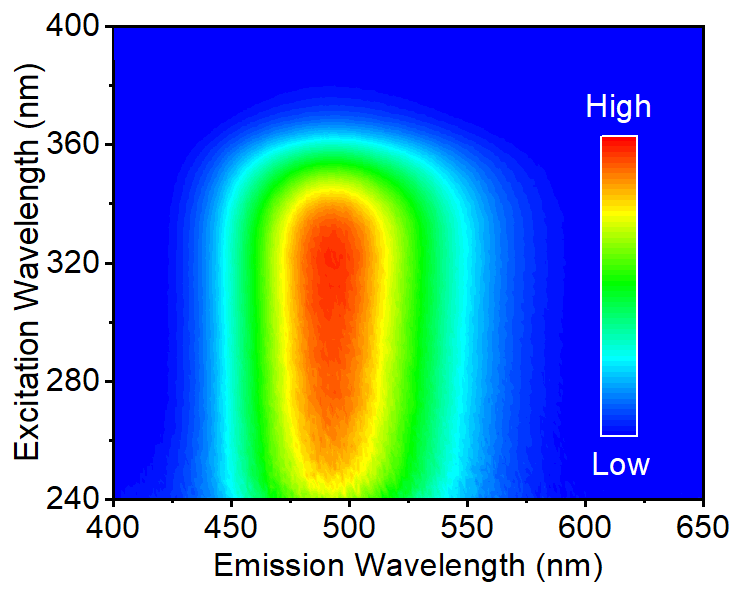


Figure S6. PL and PLE spectra of (Cs_8_Cu)Y_3_Cl_18_ at room temperature. Only one emission center could be observed from the excitation and emission contour mapping.


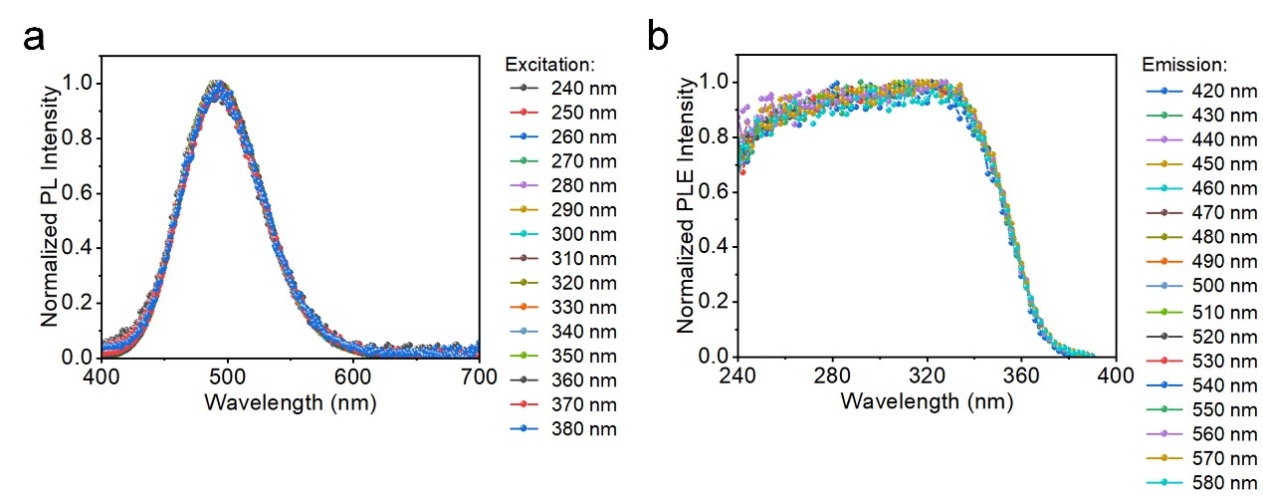


Figure S7. a, Excitation wavelength-dependent (240-380 nm) PL spectra of (Cs_8_Cu)Y_3_Cl_18_. b, Emission wavelength-dependent PLE spectra of (Cs_8_Cu)Y_3_Cl_18_. The normalized PLE (240-400 nm) and PL (400-700 nm) spectra exhibit identical shapes, revealing that the bright cyan emission originate from the radiative decay of a single center.^6^


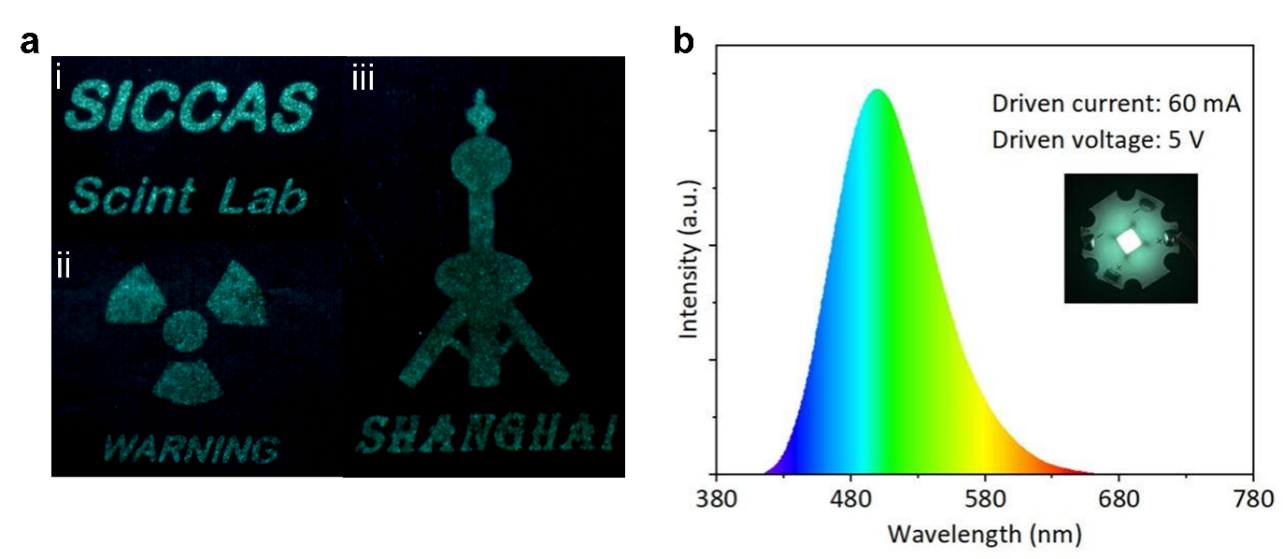


**Figure S8. Proof-of-concept demonstration of luminescence-based applications using Cs_8_CuY_3_Cl_18_. a,** Anti-counterfeiting application. Words and images printed on white A4 paper and visualized under UV irradiation, including (i) ‘’SICCAS” and “Scint Lab”, (ii) a “radiation warning” pattern, and (iii) the “Oriental Pearl Tower”. **b,** Light-emitting diode (LED) application. Representative electroluminescence (EL) spectrum of a Cs₈CuY₃Cl₁₈-based LED measured under a driving current of 60 mA and a driving voltage of 5 V. The inset shows the as-fabricated device operating at 60 mA.

Benefiting from the extremely large Stokes shift, self-absorption in (Cs_8_Cu)Y_3_Cl_18_ is effectively minimized, rendering it a highly efficient cyan-emitting phosphor.^26^ Accordingly, an all-solid-state light source was fabricated by coating (Cs_8_Cu)Y_3_Cl_18_ onto a 310 nm UV LED chip. The corresponding luminescence spectra are shown in Fig. S22, and bright cyan electroluminescence can be clearly observed from the as-fabricated device. Owing to its cyan emission, (Cs_8_Cu)Y_3_Cl_18_ phosphors can compensate for the spectral valley between the blue and yellow emission bands, thereby improving the color rendition of phosphor-converted LEDs (pc-LEDs).^27^ In addition, the outstanding thermal stability of (Cs_8_Cu)Y_3_Cl_18_ ensures its reliable long-term operation in solid-state lighting applications.^28^ Furthermore, its potential applications in anti-counterfeiting and display were preliminarily explored using a facile silk-screen printing method. As a proof-of-concept demonstration, the words “SICCAS” and “Scint Lab”, as well as the patterns of a “radiation warning” symbol and the “Oriental Pearl Tower”, were printed on white A4 paper (Fig. S8). Under UV irradiation, these patterns exhibit bright and vivid cyan–green emission, highlighting the multifunctional luminescence potential of (Cs_8_Cu)Y_3_Cl_18_ for applications such as anti-forgery, information security, and brand protection.^29, 30^


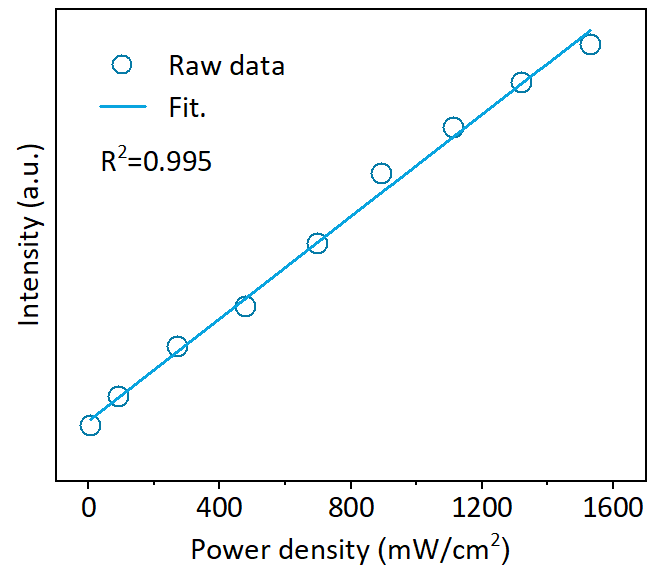


Figure S9. Emission intensity at 490 nm under various excitation power of (Cs_8_Cu)Y_3_Cl_18_.


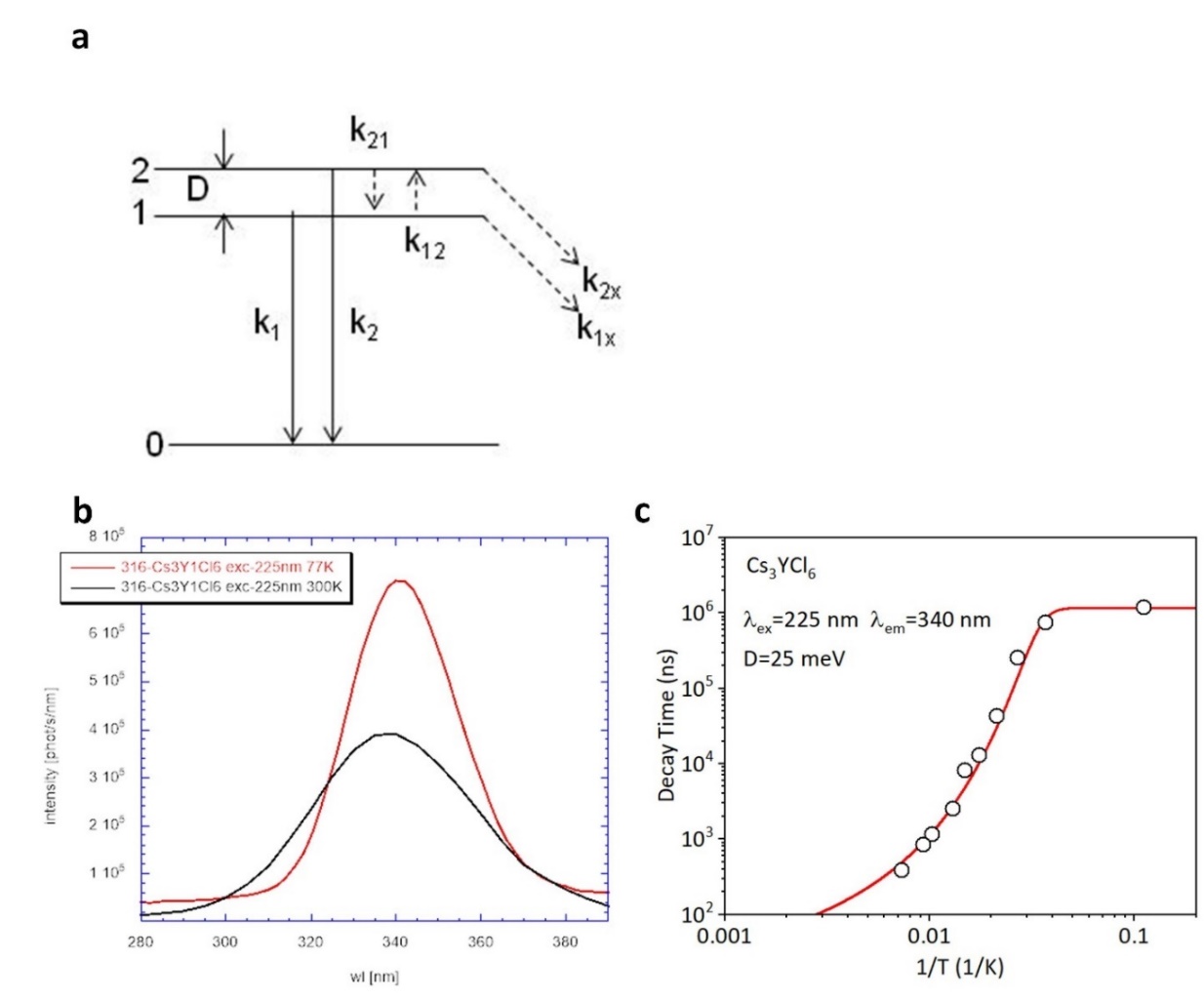


Figure S10. a, The scheme of the two-excited-level model. b, PL spectra of Cs_3_YCl_6_ measured at room temperature and 77 K. c, Temperature dependence of PL decay times of the 340 nm emission under the 225 nm excitation in Cs_3_YCl_6_. Empty circles are experimental data, solid line is the best fit of the model in (a) to the data.

Temperature dependence of PL spectra and decay of Cs_3_YCl_6_ are displayed in **Figure S9b,c.** As the excitation spectrum of 340 nm emission shows the low energy band situated within 220-250 nm well separated from the absorption edge with an onset probably around 200 nm (**Figure 2A**), at 225 nm we more likely excite a defect state, rather than an exciton.

The temperature dependence of PL decay times of the 340 nm emission in Cs_3_YCl_6_ can be described within the two-excited-level model (schematically shown in **Fig. S9a**), representing the structured relaxed excited state (RES) of the luminescence center. Such model has been used to describe the excited-state dynamics of heavy ns^2^ ions (Tl^+^, Pb^2+^ or Bi^3+^) in various hosts, see, e.g. reference,**^31^**. The time evolution of the populations $N_{1}, N_{2}$ of the excited levels 1 and 2, respectively, can be evaluated by the following rate equations:

$$\frac{{dN}_{1}}{dt}=-k_{1}N_{1}-k_{12}N_{1}+k_{21}N_{2}-k_{1x}N_{1}$$

$$\frac{{dN}_{2}}{dt}=-k_{2}N_{2}-k_{21}N_{2}+k_{12}N_{1}-k_{2x}N_{2} , (1)$$

where $k_{1}, k_{2}, k_{12}, k_{21},$ and $k_{1(2)x}$ are radiative transition rates from levels 1,2, non-radiative rates of phonon assisted transitions between the radiative level 2 and metastable level 1 and the quenching channel from the level 1(2), respectively. Non-radiative transitions between levels 1,2 can be written as:

$k_{21}=K\left( n+1 \right), k_{12}=Kn,$

$n=1/{[\exp\left( D/{k_{B}T} \right)-1]}$. $(2)$

Here $K, n, D$ are the zero-temperature transition rate between the levels 1 and 2, the Bose-Einstein factor and energy spacing between the levels, respectively. Non-radiative quenching channel, if present, is considered in the usual barrier form:

$k_{1(2)x}=K_{1\left( 2 \right)x}\left( -\frac{E_{1(2)x}}{k_{B}T} \right)$ $(3)$

with *K*_1(2)x_ being a frequency factor and *E*_1(2)x_ the height of the barrier. The model yields a decay with two components, fast and slow. However, only slow decay component was experimentally measurable (empty circles in Fig. 2c). The intensity of emission upon 225 nm excitation does not change much when 77 K and room temperature are compared (Fig. S9b), so that there is no significant thermal quenching at room temperature (RT) and the acceleration of the slow component decay time in Fig. S9c at RT is given by thermal population of level 2 at higher temperatures which is significantly more allowed, see the energy level diagram in Fig. S9a and the transition rate $\boldsymbol{k}_{\boldsymbol{12}}$. The best fit of the model to the data without employing the quenching channels described by (3) is reported by the solid line in Fig. S9c. Characteristic parameters of the model (Fig. S9a) used in the best fit (Fig. S9c) are k_1_=850 s^-1^; k_2_=7×10^7^ s^-1^; K=2×10^7^ s^-1^; D=25 meV.

However, TD of decay times measured under the excitation at 200 nm in **Fig. 2c** is considerably different compared to the case λ_ex_=225 nm, likely due to different nature of the luminescent center. Additionally, a quenching pathway has to be added from the level 1 (K_1x_, E_x_) to satisfactorily fit the data at the highest temperatures. Consequently, the binding energy of exciton could be close to E_1x_=170 meV upon the assumption that the quenching is due to the exciton thermal disintegration (**Fig. 2c** and **S9a**).


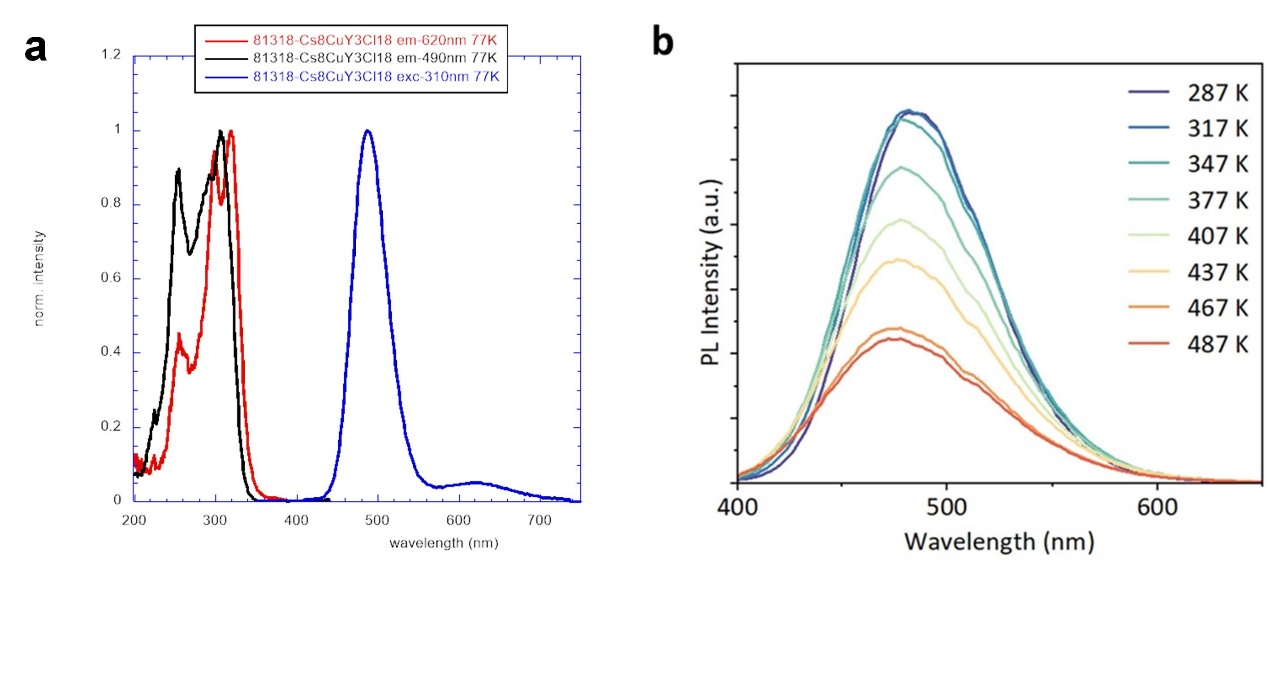


Figure S11. a, PL and PLE spectra of (Cs_8_Cu)Y_3_Cl_18_ at 77 K. b, Temperature dependence of PL spectra in (Cs_8_Cu)Y_3_Cl_18_.


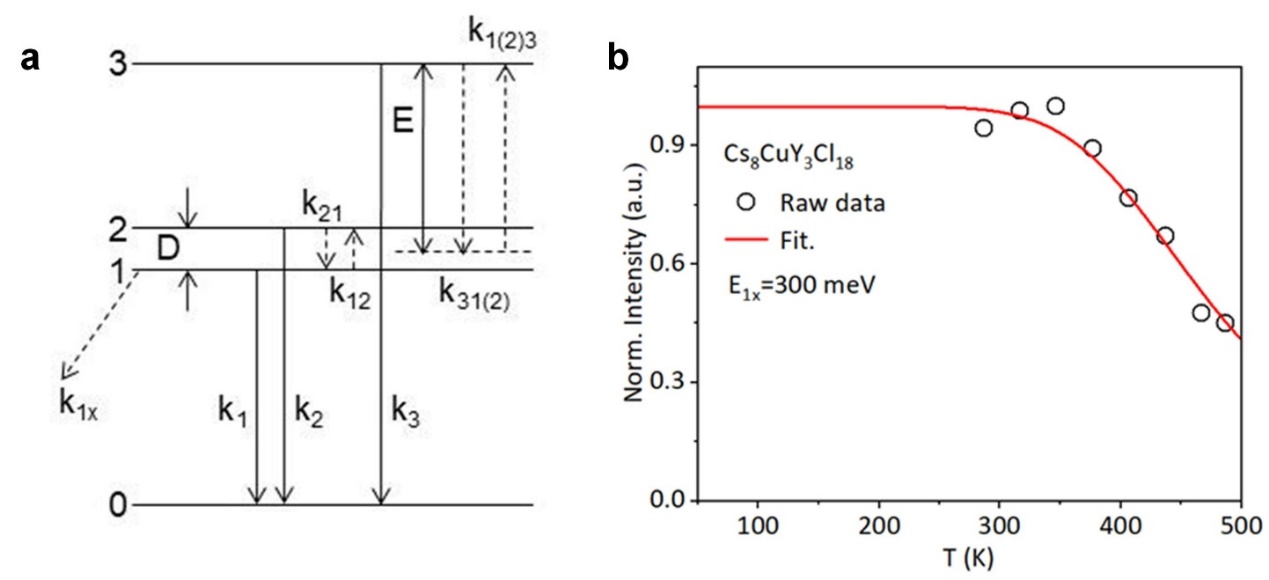


Figure S12. a, Three-excited-level model used to fit the temperature dependence of both the STE decay times and intensity in (Cs_8_Cu)Y_3_Cl_18_. b, Temperature dependence of PL emission intensity of the center I (490 nm) in (Cs_8_Cu)Y_3_Cl_18_. Empty circles are experimental data, solid line is the best fit of the model in (a) to the data.

Temperature dependence of PL decay times of (Cs_8_Cu)Y_3_Cl_18_ displayed in **Fig. 2F** can be described within the three-excited-level model (schematically shown in **Fig. S11a**), representing the structured RES of the STE with lower levels 1,2, corresponding to the split triplet state and the upper level 3 corresponding to the singlet state of STE. Such model has been used to describe the excited-state dynamics of STE in halides or oxides.^32^ The time evolution of the populations $N_{1}, N_{2}, N_{3}$ of the excited levels 1,2 and 3, respectively, can be evaluated by the following rate equations:

$$\frac{{dN}_{1}}{dt}=-k_{1}N_{1}-k_{12}N_{1}-k_{13}N_{1}+k_{21}N_{2}+k_{31}N_{3}-k_{1x}N_{1}$$

$$\frac{{dN}_{2}}{dt}=-k_{2}N_{2}-k_{21}N_{2}-k_{23}N_{2}+k_{12}N_{1}+k_{32}N_{3} \left( 4 \right)$$

$$\frac{{dN}_{3}}{dt}=-k_{3}N_{3}-k_{31}N_{3}-k_{32}N_{3}+k_{13}N_{1}+k_{23}N_{2} ,$$

where analogous parameters have the same meaning as in (1). In addition, $k_{3}$is the radiative transition rate from the singlet level 3 and $k_{3(2)1}$, $k_{1(2)3}$are non-radiative transitions between levels 3(2) and 1:

$k_{31}=K'\left( n^{'}+1 \right), k_{13}=K'n',$

$n'=1/{[\exp\left( E/{k_{B}T} \right)-1]}$. $(5)$

Here $K^{'}, n^{'}$ have analogous meaning as $K, n$ and $E$ is the energy spacing between the singlet and triplet levels. Since $D\ll E$ we consider

$k_{31}=k_{32} , k_{13}=k_{23} (6)$

The model yields a decay with three components. However, only two decay components were experimentally measurable (solid circles in Fig. 2f). The fastest decay component is frequently experimentally not observed due to low initial population of the upper state that results in its too low prompt intensity. However, consideration of the level 3 in the model is essential to explain the temperature dependence of other two slower components due to nonradiative channels involving level 3 in depopulation of levels 1,2.

The best fit of the model to the decay time data and intensity employing the quenching channel from the level 1 described by (3) is reported by the solid lines in Fig. 2f and Fig. S12b, respectively.


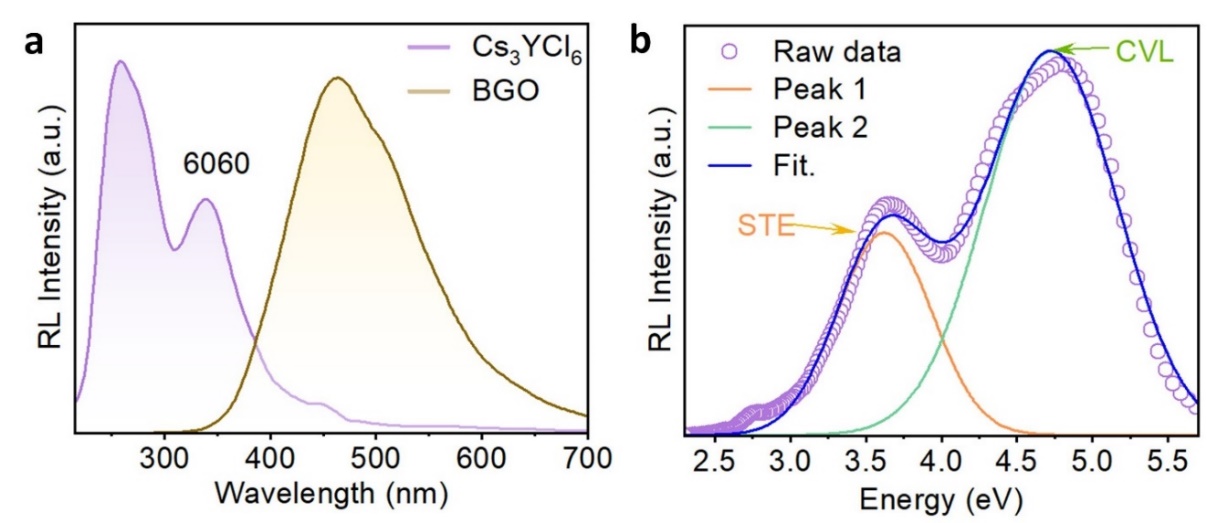


Figure S13. a, X-ray-excited RL spectrum of Cs_3_YCl_6_ and BGO under identical measurement conditions. b, The emission peak Gaussian fitting of Cs_3_YCl_6_. The RL spectra of Cs_3_YCl_6_ are composed by the STE and by the core-valence luminescence (CVL) emissions. The scintillation yield of Cs_3_YCl_6_ is calculated to be 6,440 photons MeV^-1^ by comparing its integral area of RL spectrum with that of BGO (8,500 photons MeV^-1^) using an integrating sphere. RL spectra are measured under the same experimental conditions (the sample’s shape, reflection geometry, sufficient thickness about 1mm to absorb all the energy from X-ray excitation). The measured RL spectra are recalculated using appropriate calibration curves to remove experimental distortion.


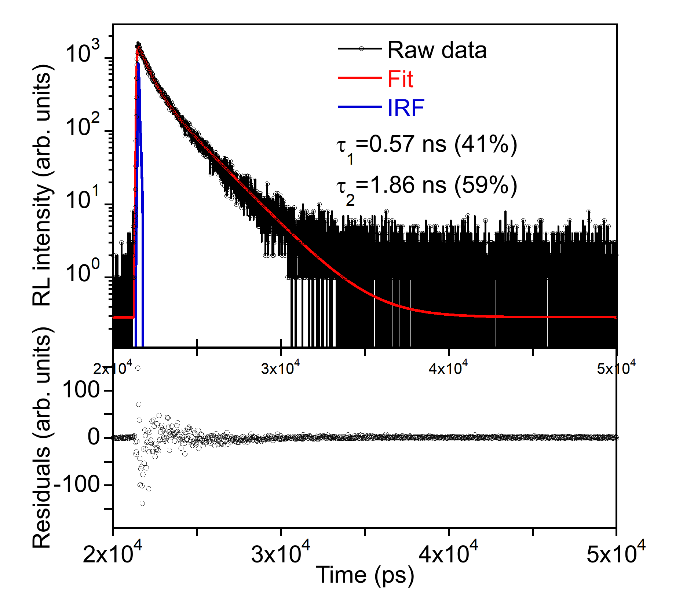


Figure S14. Scintillation decay profiles of Cs_3_YCl_6_ under pulse X-ray excitation at room temperature.

In the short time window (50 ns), a very fast decay was registered. The instrumental response function (IRF) full width at half maximum of the setup is about 75 ps. A convolution procedure was applied to the decay curve to determine the true decay times (SpectraSolve^TM^ software package, Ames Photonics, USA). The decay profiles can be well fitted by two components, 0.57 ns (41%) and 1.86 ns (59%). The residuals indicate a good fit without non-random patterns, and the goodness-of-fit metrics chi-square (χ^2^) value was 1.34. This process matches the expected CVL time characteristics, i.e. fast radiative electronic transition between the valence band and the outermost core band. Reliable slow decay was not obtained over longer time windows because it was too slow or too weak for the set up. This further confirms the 3.5-5.5 eV emission attributed to CVL luminescence.


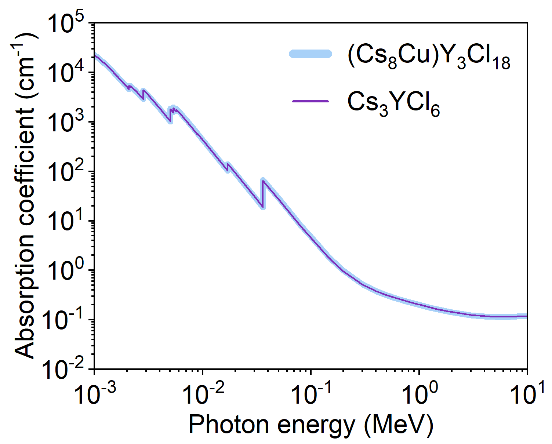


Figure S15. Absorption coefficients of (Cs_8_Cu)Y_3_Cl_18_ compared with Cs_3_YCl_6_.


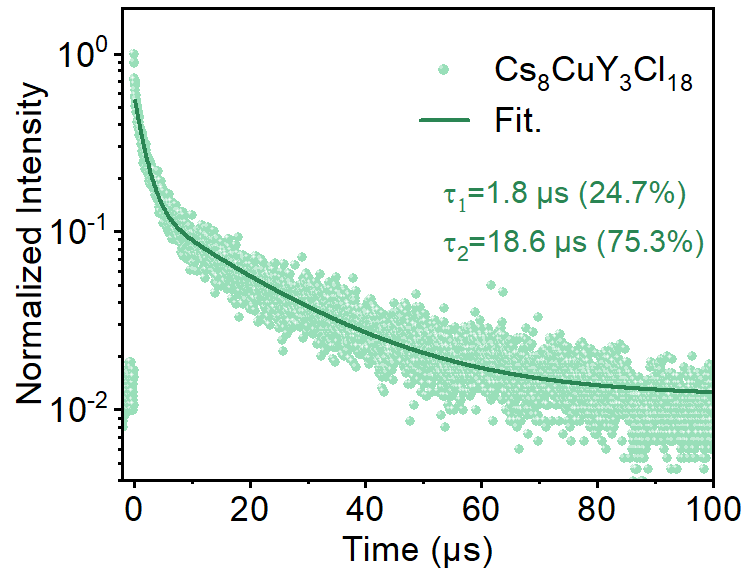


Figure S16. Scintillation decay profiles of (Cs_8_Cu)Y_3_Cl_18_ under pulse X-ray excitation at room temperature.


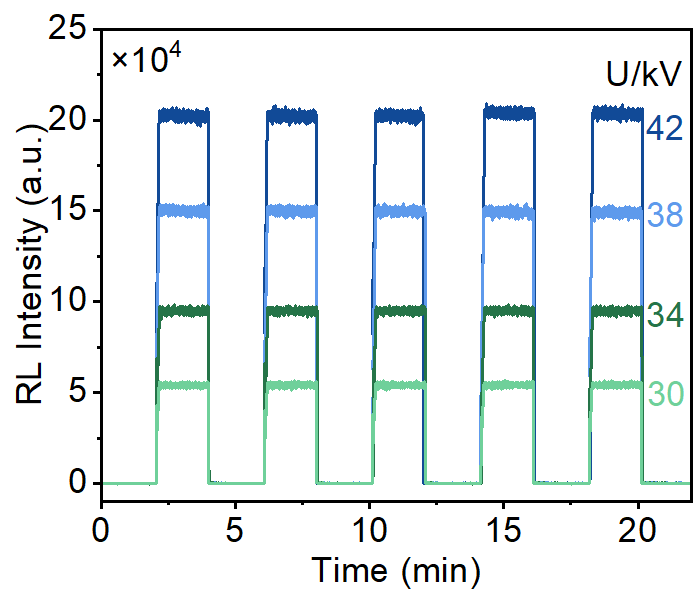


Figure S17. The radiation stability of (Cs_8_Cu)Y_3_Cl_18_ under cyclical X-ray irradiation.


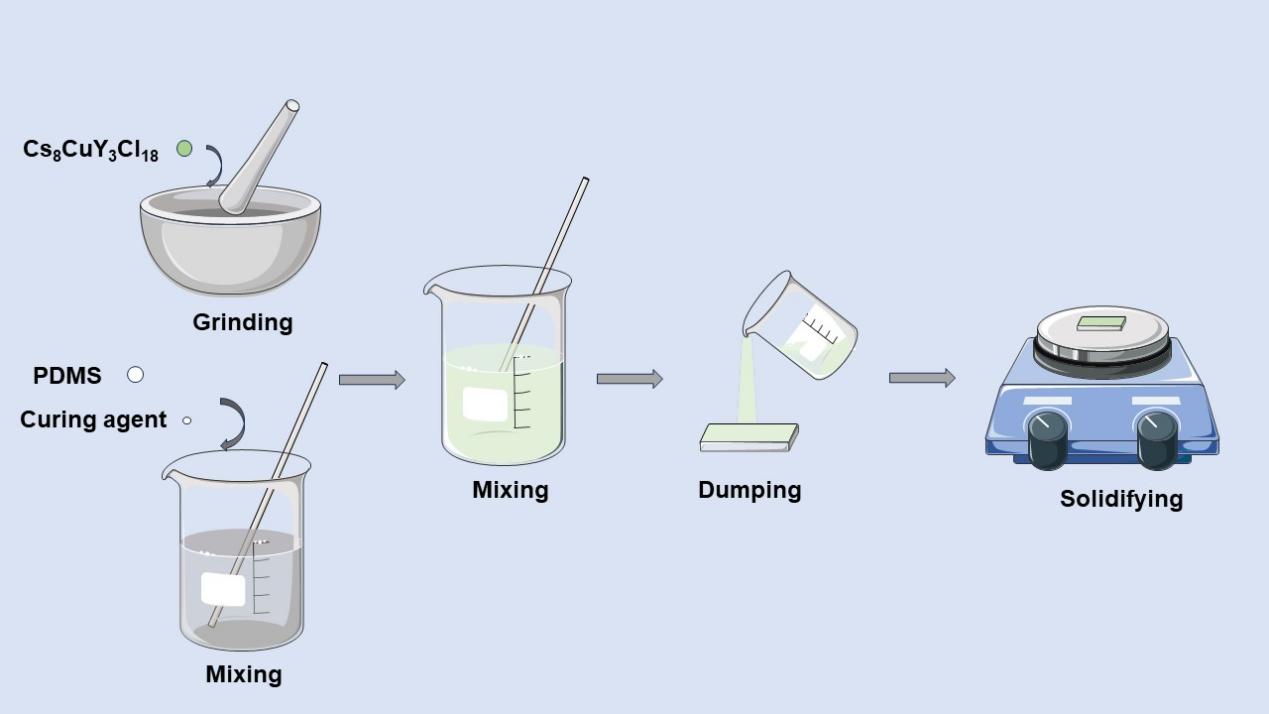


Figure S18. Schematic preparation of the (Cs_8_Cu)Y_3_Cl_18_@PDMS composite scintillation films.


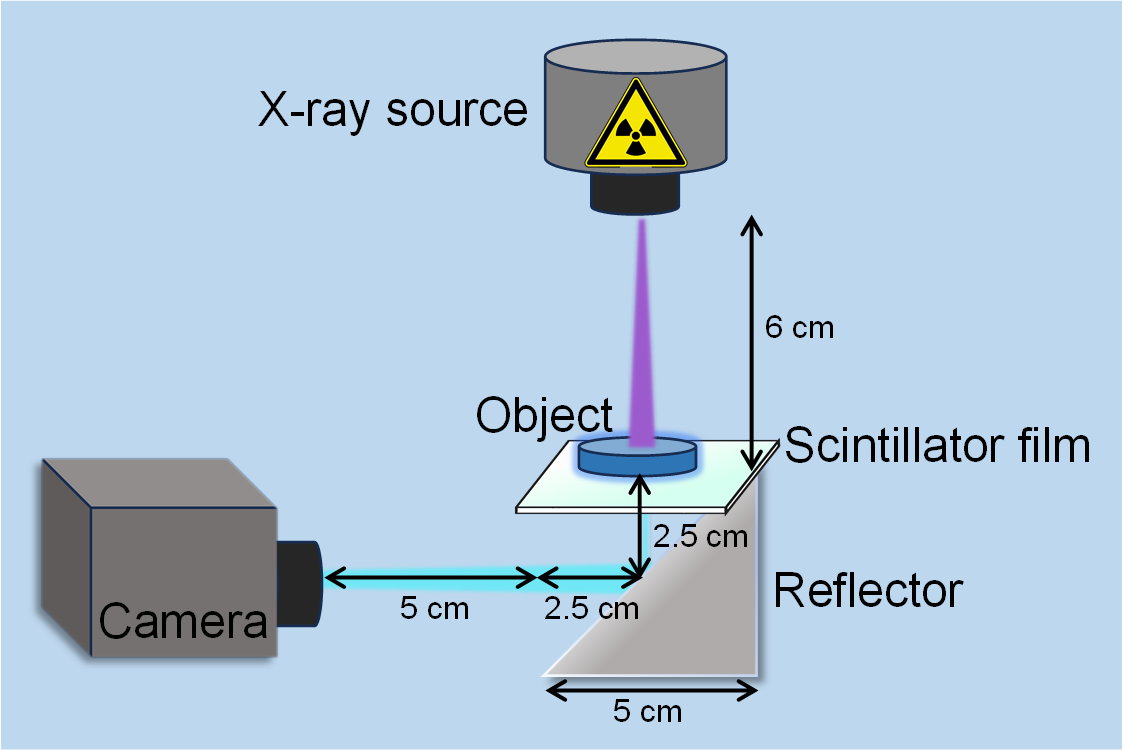


Figure S19. Schematic diagram of the X-ray phase-contrast imaging system.


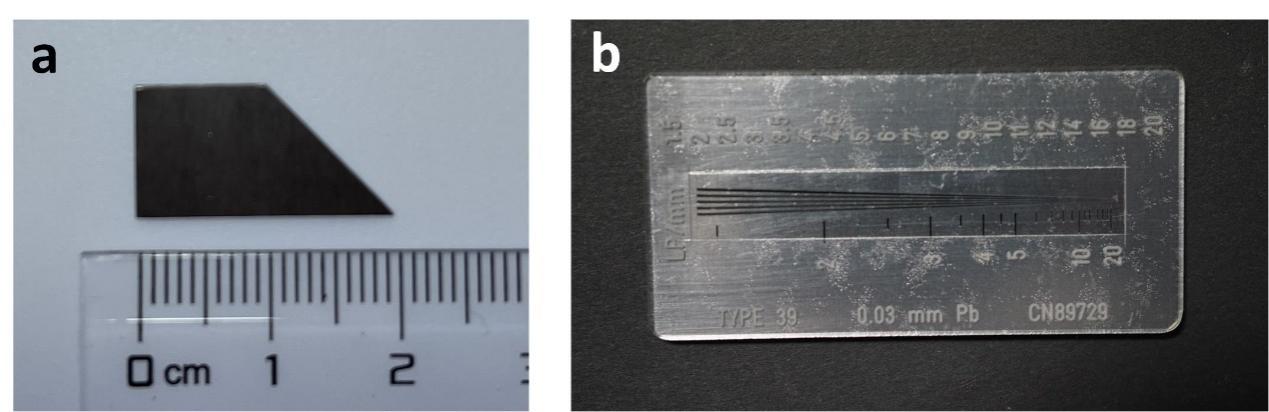


Figure S20. a, Image of 1 mm thick tungsten slide used in MTF calculation. b, Image of standard X-ray resolution pattern plate (0.3 mm Pb, Type 39).


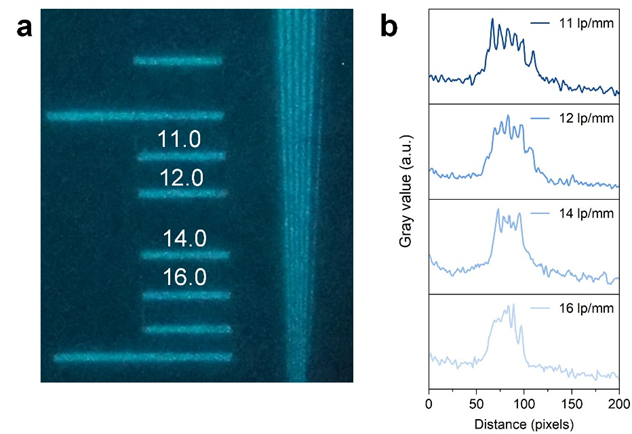


**Figure S21. X-ray imaging spatial-resolution validation using a standard line-pair target. a,** X-ray image acquired using the (Cs_8_Cu)Y_3_Cl_18_ scintillation screen for a clearly identified partial region (12-14 lp mm⁻¹) of a standard X-ray line-pair card. The image was recorded under the same experimental conditions as those used for the slanted-edge MTF measurements, including identical X-ray excitation, optical collection geometry, and detector settings. **b,** Corresponding grayscale intensity profiles extracted perpendicular to the line-pair direction. The periodic modulation of the gray value demonstrates that individual line pairs remain distinguishable up to ~14 lp mm⁻¹, providing a direct visibility-based criterion for spatial resolution. The line-pair-based assessment is consistent with the spatial resolution of 14.6 lp mm⁻¹ at MTF = 0.2 derived from the tungsten-edge method.

**
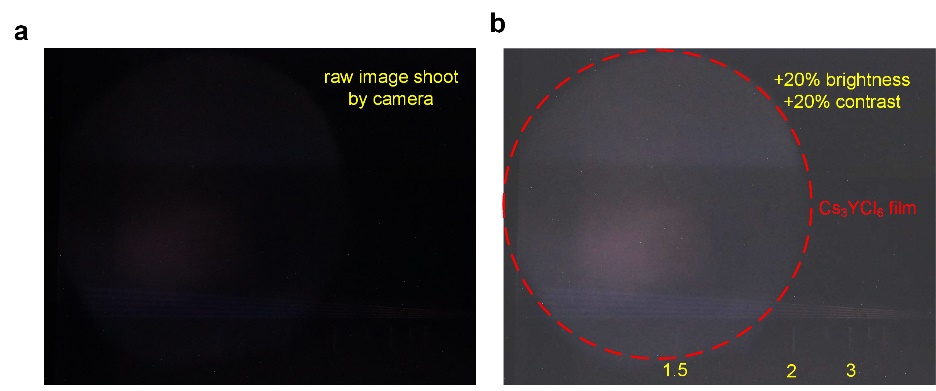
**

Figure S22. X-ray images based on Cs_3_YCl_6_ scintillation screen of a partial region (1.5-2 lp mm^−1^) of the standard X-ray line pair card.

The X-ray images of Cs_3_YCl_6_ and (Cs_8_Cu)Y_3_Cl_18_ were collected using identical imaging parameters and geometry to ensure a fair comparison of imaging performance. However, due to the weak STE-related scintillation yield of Cs_3_YCl_6_, clear line-pair images could not be obtained under the same imaging conditions used for (Cs_8_Cu)Y_3_Cl_18_, which prevents a reliable determination of spatial resolution directly from the raw image. To provide a more intuitive comparison of imaging performance between Cs_3_YCl_6_ and (Cs_8_Cu)Y_3_Cl_18_, we increased the image brightness and contrast by 20% during post-processing. Even after this adjustment, the spatial resolution of the Cs_3_YCl_6_@PDMS film can only be approximately estimated to be 1.5-2 lp mm^-1^ (Fig. S22), which is substantially lower than that of (Cs_8_Cu)Y_3_Cl_18_. This difference originates from the significantly lower scintillation efficiency of Cs_3_YCl_6_ rather than imaging focus or acquisition conditions.


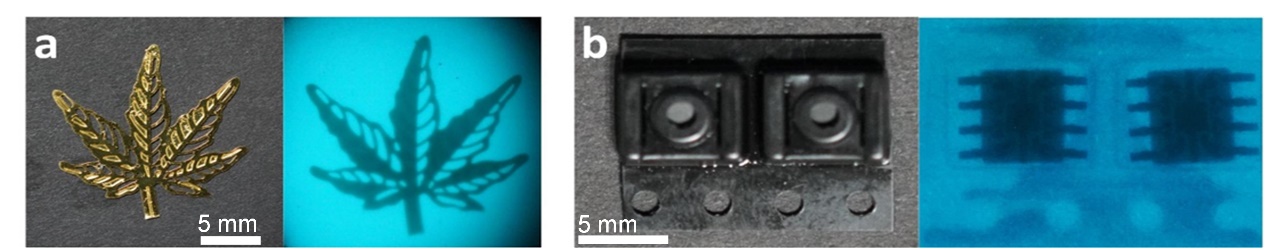


Figure S23. X-ray imaging demonstrations obtained by using the (Cs_8_Cu)Y_3_Cl_18_ scintillation screen. a, Image of a metallic flower under bright-field (left) and X-ray (right). The scale bar is 5 mm. b, Image of a micro-chip under bright-field (left) and X-ray (right). The scale bar is 5 mm.

**
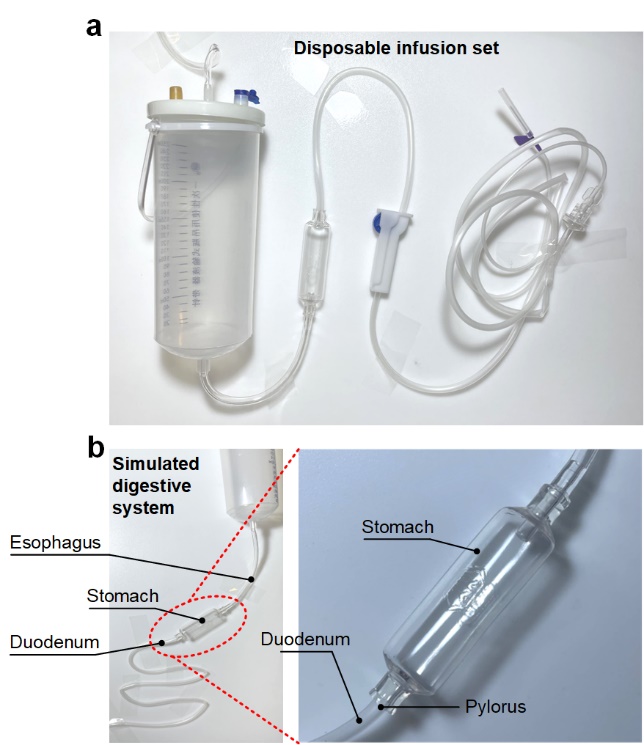
**

Figure S24. a, Image of the disposable infusion set. b, Corresponding simulated organs of upper digestive system in the barium meal examination.


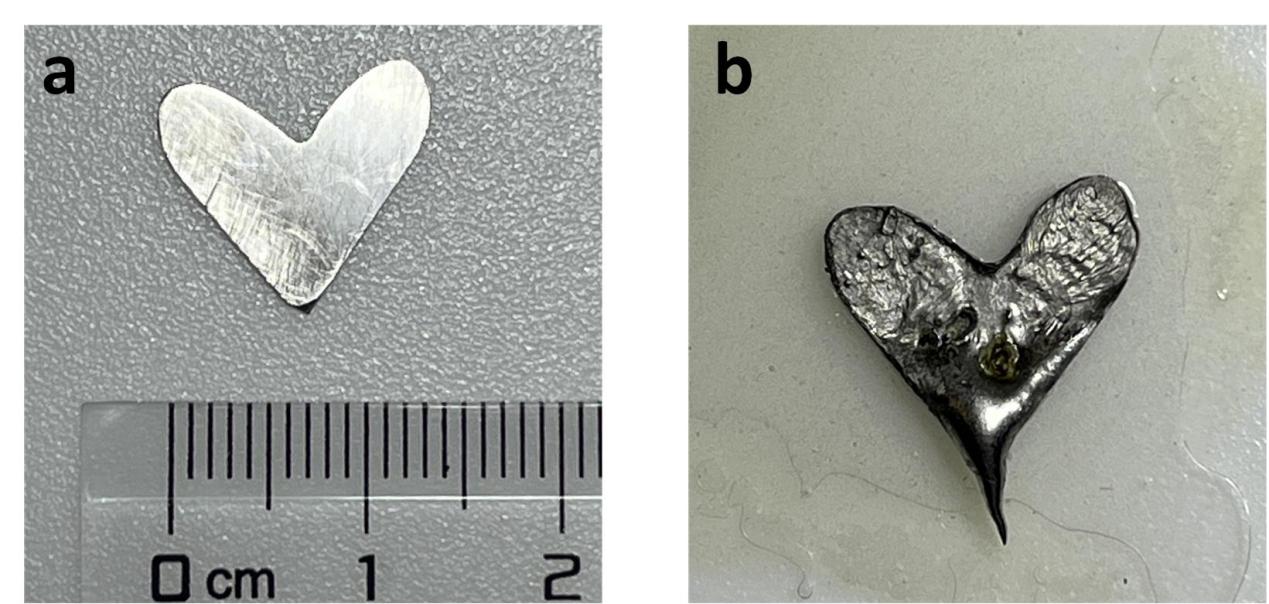


Figure S25. a, Image of bismuth-indium-tin alloy (1 mm thick, melting point of 423 K) used in stimulate the high-temperature X-ray imaging application. b, Image of melted bismuth-indium-tin alloy after been heated.

Table S1. Single crystal X-ray diffraction data and structure refinement of (Cs_8_Cu)Y_3_Cl_18_ and Cs_3_YCl_6_.

| Formula | (Cs_8_Cu)Y_3_Cl_18_ | Cs_3_YCl_6_ |
| --- | --- | --- |
| Formula weight | 8126.60 | 5602.72 |
| Temperature/K | 100 | 293(2) |
| Radiation, wavelength/Å | Mo Ka, 0.71073 | Mo Ka, 0.71073 |
| Crystal system | hexagonal | monoclinic |
| Space group | P6_3_/m | C2/c |
| a/ Å | 13.1463(2) | 26.6879(9) |
| b/ Å | 13.1463(2) | 8.1113(3) |
| c/ Å | 26.4252(4) | 12.9323(4) |
| α/° | 90 | 90 |
| β/° | 90 | 99.778(3) |
| γ/° | 120 | 90 |
| Volume/ Å^3^ | 3955.53(14) | 2758.83(16) |
| Density/g cm^-3^ | 3.412 | 3.372 |
| 2θ range for data collection/° | 1.789-26.370 | 2.628-26.371 |
| Reflections collected | 66044 | 29363 |
| Independent reflections | 2736 | 2816 |
| Observed reflections | 2735 | 2811 |
| restraints/parameters | 0/96 | 0/93 |
| R_obs_,wR_obs_ | 0.0321, 0.0620 | 0.0786, 0.1579 |
| R_all_,wR_all_ | 0.0321, 0.0620 | 0.0787, 0.1579 |
| Goodness-of-fit on F^2^ | 1.699 | 1.515 |

Table S2. Bond lengths (Å) for (Cs_8_Cu)Y_3_Cl_18_.

| Cs1 | Cs2 | 4.6235(3) | Cs3 | Cl5 | 3.5125(14) |
| --- | --- | --- | --- | --- | --- |
| Cs1 | Cs3 | 4.5906(3) | Cs3 | Cl5 | 3.5126(14) |
| Cs1 | Y3 | 4.8075(7) | Cs4 | Y2 | 4.5501(5) |
| Cs1 | Cu1 | 4.0752(4) | Cs4 | Cl3 | 4.0008(15) |
| Cs1 | Cl1 | 3.4653(13) | Cs4 | Cl4 | 3.6213(14) |
| Cs1 | Cl2 | 3.4543(13) | Cs4 | Cl5 | 3.7090(15) |
| Cs1 | Cl3 | 3.5369(14) | Cs4 | Cl5 | 3.9380(15) |
| Cs1 | Cl3 | 3.5170(14) | Cs4 | Cl5 | 3.6551(14) |
| Cs1 | Cl4 | 3.3941(14) | Cs4 | Cl6 | 3.4660(14) |
| Cs1 | Cl5 | 3.5086(13) | Cs4 | Cl6 | 3.6430(15) |
| Cs1 | Cl6 | 3.3047(13) | Cs4 | Cl6 | 3.8148(15) |
| Cs1 | Cl7 | 3.4084(13) | Cs4 | Cl7 | 4.0221(15) |
| Cs2 | Cs2 | 4.3954(12) | Cs4 | Cl7 | 3.6928(15) |
| Cs2 | Y2 | 4.2555(10) | Cs4 | Cl7 | 3.7127(14) |
| Cs2 | Cl1 | 3.4790(15) | Y1 | Cl5 | 2.6246(13) |
| Cs2 | Cl1 | 3.4789(15) | Y1 | Cl5 | 2.6246(13) |
| Cs2 | Cl1 | 3.4789(15) | Y1 | Cl5 | 2.6247(13) |
| Cs2 | Cl4 | 3.8939(15) | Y1 | Cl5 | 2.6246(13) |
| Cs2 | Cl4 | 3.8939(15) | Y1 | Cl5 | 2.6246(13) |
| Cs2 | Cl4 | 3.8939(15) | Y1 | Cl5 | 2.6246(13) |
| Cs2 | Cl7 | 3.4593(14) | Y2 | Cl6 | 2.5926(14) |
| Cs2 | Cl7 | 3.4594(14) | Y2 | Cl6 | 2.5926(14) |
| Cs2 | Cl7 | 3.4593(14) | Y2 | Cl6 | 2.5926(14) |
| Cs3 | Cs3 | 4.5529(12) | Y2 | Cl7 | 2.6337(14) |
| Cs3 | Y1 | 4.3331(6) | Y2 | Cl7 | 2.6336(14) |
| Cs3 | Cl2 | 3.5026(15) | Y2 | Cl7 | 2.6337(14) |
| Cs3 | Cl2 | 3.5025(15) | Y3 | Cl1 | 2.628(2) |
| Cs3 | Cl2 | 3.5025(15) | Y3 | Cl2 | 2.628(2) |
| Cs3 | Cl4 | 3.8574(15) | Y3 | Cl3 | 2.6926(14) |
| Cs3 | Cl4 | 3.8574(15) | Y3 | Cl3 | 2.6926(14) |
| Cs3 | Cl4 | 3.8574(15) | Y3 | Cl4 | 2.5798(14) |
| Cs3 | Cl5 | 3.5126(14) | Y3 | Cl4 | 2.5798(14) |
| Cu1 | Cl3 | 2.2551(14) | Cu1 | Cl3 | 2.2551(14) |
| Cu1 | Cl3 | 2.2552(14) |  |  |  |

Table S3. Temperature dependence of PL decay times within 77-487 K at 490 nm in (Cs_8_Cu)Y_3_Cl_18_.

| Temperature (K) | Component 1 (µs) | Component 2 (µs) |
| --- | --- | --- |
| 77 | 45.68 (97.6%) | 380.4 (2.4%) |
| 107 | 43.54 (97.1%) | 379.4 (2.9%) |
| 137 | 40.42 (96.1%) | 291 (3.9%) |
| 167 | 39.18 (95.5%) | 239.7 (4.5%) |
| 197 | 39.04 (95.1%) | 191 (4.9%) |
| 227 | 39.52 (95.3% | 167.1 (4.7%) |
| 257 | 37.26 (95.4%) | 145.7 (4.6%) |
| 287 | 36.14 (95.5%) | 120.4 (4.5%) |
| 317 | 35.55 (96.8%) | 111.2 (3.2%) |
| 347 | 34.78 (97.8%) | 100.2 (2.2%) |
| 377 | 28.13 (72.2%) | 41.48 (27.8%) |
| 407 | 31.04 (83.7% | 25.79 (16.3%) |
| 437 | 27.32 (77.7%) | 34.25 (22.3%) |
| 467 | 28.73 (83.8%) | 24.45 (16.2%) |
| 487 | 26.92 (88.6%) | 30.78 (11.4%) |

**Table S4**. Comparison of spatial resolution and detection limit of PDMS-based scintillation films.

| Materials | Spatial resolution | Detection limit | Reference |
| --- | --- | --- | --- |
| Cs_6_Cu_3_AgBr_10_^26^ | 8.06 lp mm^−1^ | 45.9 nGy s^−1^ | 27 |
| [DDPACDBFDP]_2_Cu_4_I_4_^33^ | 12 lp mm^−1^ | 77 nGy s^−1^ | 28 |
| Cs_3_Cu_2_I_5_:Zn^34^ | 13.4 lp mm^−1^ | 178.7 nGy s^−1^ | 29 |
| Rb_2_AgBr_3_:Cu^35^ | 5.8 lp mm^−1^ | 714.83 nGy s^−1^ | 30 |
| (C_38_H_34_P_2_)MnBr_4_^36^ | - | 461.1 nGy s^−1^ | 31 |
| Cs_2_CdBr_2_Cl_2_:5%Mn^37^ | 12.3 lp mm^−1^ | 17.82 nGy s^−1^ | 32 |
| BA_2_PbBr_4_:10% Mn^38^ | 10.7 lp mm^−1^ | 16 nGy s^−1^ | 33 |
| Cs_2_ZnBr_4_:25%Mn^39^ | 5.06 lp mm^−1^ | 1.16 μGy s^−1^ | 34 |
| **Cs_3_YCl_6_** | **1.5-2 lp mm^−1^** | **-** | **This work** |
| **(Cs_8_Cu)Y_3_Cl_18_** | **14.6 lp mm^−1^** | **147 nGy s^−1^** | **This work** |

**Supplementary Reference**

1. Wu H, Wang Q, Zhang A, Niu G, Nikl M, Ming C*, et al.* One-dimensional scintillator film with benign grain boundaries for high-resolution and fast X-ray imaging. *Science Advances* 2023, 9(30): eadh1789.

2. Cheng SL, Beitlerova A, Kucerkova R, Mihokova E, Nikl M, Zhou ZY*, et al.* Non-Hygroscopic, Self-Absorption Free, and Efficient 1D CsCu_2_I_3_ Perovskite Single Crystal for Radiation Detection. *ACS Appl Mater Interfaces* 2021, 13(10): 12198-12202.

3. He Q, Zhou C, Xu L, Lee S, Lin X, Neu J*, et al.* Highly Stable Organic Antimony Halide Crystals for X-ray Scintillation. *ACS Materials Letters* 2020, 2(6): 633-638.

4. Zaffalon ML, Wu Y, Cova F, Gironi L, Li X, Pinchetti V*, et al.* Zero‐Dimensional Gua_3_SbCl_6_ Crystals as Intrinsically Reabsorption‐Free Scintillators for Radiation Detection. *Advanced Functional Materials* 2023, 33(48): 2305564.

5. Cheng S, Nikl M, Beitlerova A, Kucerkova R, Du X, Niu G*, et al.* Ultrabright and highly efficient all‐inorganic zero‐dimensional perovskite scintillators. *Advanced Optical Materials* 2021, 9(13): 2100460.

6. Wang SJ, Liu RZ, Li JC, Sun FK, Yang Q, Li S*, et al.* Achieving Highly Efficient Warm‐White Light Emission in All‐Inorganic Copper‐Silver Halides via Structural Regulation. *Adv Sci* 2023, 10(28): 2303501.

7. Cui H, Zhu W, Deng Y, Jiang T, Yu A, Chen H*, et al.* Lead‐free organic–inorganic hybrid scintillators for X‐ray detection. *Aggregate* 2023, 5(2): e454.

8. Burger A, Rowe E, Groza M, Figueroa KM, Cherepy NJ, Beck PR*, et al.* Cesium hafnium chloride: A high light yield, non-hygroscopic cubic crystal scintillator for gamma spectroscopy. *Appl Phys Lett* 2015, 107(14): 143505.

9. Li S, Zhao Y, Du L, He Y, Wang R, Guo Y*, et al.* Moisture-Assisted Molecular Sieve-Confined Synthesis of Lead-Free CsAgCl2 Perovskite-Derivative Nanocrystals. *Chemical Engineering Journal* 2024, 482: 148966.

10. Bhattacharya P, Brown C, Sosa C, Wart M, Miller S, Brecher C*, et al.* Tl₂ZrCl₆ and Tl₂HfCl₆ Intrinsic Scintillators for Gamma Rays and Fast Neutron Detection. *IEEE Transactions on Nuclear Science* 2020, 67(6): 1032-1034.

11. Kodama S, Kurosawa S, Morishita Y, Usami H, Torii T, Hayashi M*, et al.* Growth and Scintillation Properties of a New Red-Emitting Scintillator Rb₂HfI₆ for the Fiber-Reading Radiation Monitor. *IEEE Transactions on Nuclear Science* 2020, 67(6): 1055-1062.

12. Morad V, Shynkarenko Y, Yakunin S, Brumberg A, Schaller RD, Kovalenko MV. Disphenoidal Zero-Dimensional Lead, Tin, and Germanium Halides: Highly Emissive Singlet and Triplet Self-Trapped Excitons and X-ray Scintillation. *J Am Chem Soc* 2019, 141(25): 9764-9768.

13. Cheng H, Hu X, Cao C, Li X, Xie R, Wang D*, et al.* Zero-dimensional hybrid zinc halide scintillators with efficient blue light emission for X-ray imaging. *Journal of Alloys and Compounds* 2023, 968: 171950.

14. Hawrami R, Ariesanti E, Buliga V, Burger A, Lam S, Motakef S. Tl_2_HfCl_6_ and Tl_2_ZrCl_6_: Intrinsic Tl-, Hf-, and Zr-based scintillators. *J Crys Grow* 2020, 531: 125316.

15. Guo H. Synthesis of low-dimensional metal halide CsAgCl_2_ for X-ray scintillation applications. *J Lumin* 2025, 277, 120924.

16. Zhang Z, Guo X, Huang K, Sun X, Li X, Zeng H*, et al.* Lead-free bright yellow emissive Rb_2_AgCl_3_ scintillators with nanosecond radioluminescence. *J Lumin* 2022, 241: 118500.

17. Meng H, Chen B, Zhu W, Zhou Z, Jiang T, Xu X*, et al.* Stable Organic Antimony Halides with Near‐Unity Photoluminescence Quantum Yield for X‐Ray Imaging. *Laser & Photonics Reviews* 2023, 17(7): 2201007.

18. Wen X, Buryi M, Babin V, John D, Kučerková R, Nikl M*, et al.* Deciphering the mechanism of ultrafast scintillation in 1D silver halides. *Laser & Photonics Reviews* 2024, 18(12): 2400518.

19. Zhou Y, Wang Z, Guo L, Huang L, Liu Y, Wu M*, et al.* Ultrafast and high-resolution X-ray imaging based on zero-dimensional organic silver halides. *Materials Chemistry Frontiers* 2024,8, 3004-3016.

20. Tang Y, Pu G, Wang M, Li J, Kang C, Liu L*, et al.* Brightening dark excitons in inorganic halide perovskites by local symmetry breaking. *Materials Today Chemistry* 2024, 41, 102307.

21. Yang Q-H, Wei H-Q, Li G-H, Huang J-B, Liu X, Cai G-M. Recent developments of lead-free halide-perovskite Cs_3_Cu_2_X_5_ (X = Cl, Br, I): Synthesis, modifications, and applications. *Materials Today Physics* 2023, 36, 101143.

22. Swiderski L, Brylew K, Janiak L, Mianowska Z, Moszyński M, Mykhaylyk V*, et al.* Cs_2_ZrCl_6_ scintillation properties studied using γ-ray spectroscopy and Compton coincidence technique. *Nuclear Instruments and Methods in Physics Research Section A: Accelerators, Spectrometers, Detectors and Associated Equipment* 2023, 1057: 168735.

23. Cheng S, Beitlerova A, Kucerkova R, Nikl M, Buryi M, Shao C*, et al.* Efficient White Light‐Emitting Diodes Based on Cu(I) Activated Single‐Phase Halide Single Crystals. *physica status solidi (RRL) – Rapid Research Letters* 2024, 18(4): 2300463.

24. Nagorny S. Novel Cs_2_HfCl_6_ Crystal Scintillator: Recent Progress and Perspectives. *Physics* 2021, 3(2): 320-351.

25. Kodama S, Kurosawa S, Ohno M, Yamaji A, Yoshino M, Pejchal J*, et al.* Development of a novel red-emitting cesium hafnium iodide scintillator. *Radiation Measurements* 2019, 124: 54-58.

26. Wang S, Liu R, Li J, Sun F, Yang Q, Li S*, et al.* Achieving Highly Efficient Warm-White Light Emission in All-Inorganic Copper-Silver Halides via Structural Regulation. *Adv Sci (Weinh)* 2023, 10(28): e2303501.

27. Zhao M, Liao H, Molokeev MS, Zhou Y, Zhang Q, Liu Q*, et al.* Emerging ultra-narrow-band cyan-emitting phosphor for white LEDs with enhanced color rendition. *Light: Science & Applications* 2019, 8(1): 38.

28. Luo J, Wang X, Li S, Liu J, Guo Y, Niu G*, et al.* Efficient and stable emission of warm-white light from lead-free halide double perovskites. *Nature* 2018, 563(7732): 541-545.

29. Ma R, Wang C, Yan W, Sun M, Zhao J, Zheng Y*, et al.* Interface synergistic effects induced multi-mode luminescence. *Nano Research* 2022, 15(5): 4457-4465.

30. Yu X, Zhang H, Yu J. Luminescence anti‐counterfeiting: From elementary to advanced. *Aggregate* 2021, 2(1): 20-34.

31. Hlinka J, Mihokova E, Nikl M. Kinetics of A-Luminescence in KCl:Tl Multiphonon Processes. *Physica status solidi (b)* 1991, 166(2): 503-510.

32. Nagirnyi V, Stolovich A, Zazubovich S, Zepelin V, Mihokova E, Nikl E*, et al.* Peculiarities of the triplet relaxed excited-state structure and luminescence of a CsI:Tl crystal. *J Phys: Conden Matt* 1995, 7(18): 3637.

33. Zhang N, Qu L, Dai S, Xie G, Han C, Zhang J*, et al.* Intramolecular charge transfer enables highly-efficient X-ray luminescence in cluster scintillators. *Nature Communications* 2023, 14(1): 2901.

34. Yang Q-H, Li G-H, Ao Y-C, Zhao S-J, Wei H-Q, Fu L*, et al.* Green massive mechanical synthesis of highly efficient Zn-Doped Cs_3_Cu_2_I_5_ for LED and X-ray imaging applications. *Chemical Engineering Journal* 2024, 500: 157230.

35. Hu Y, Jin J, Han K, Xia Z. Unveiling The Role of Cu^+^ Doping in Rb_2_AgBr_3_ Scintillators toward Enhanced Photoluminescence Quantum Efficiency and Light Yield. *Adv Opt Mater* 2023, 12(90): 2302063.

36. Xu L-J, Lin X, He Q, Worku M, Ma B. Highly efficient eco-friendly X-ray scintillators based on an organic manganese halide. *Nature Communications* 2020, 11(1): 4329.

37. Xu H, Liang W, Zhang Z, Cao C, Yang W, Zeng H*, et al.* 2D perovskite Mn^2+^‐doped Cs_2_CdBr_2_Cl_2_ Scintillator for low‐dose high‐resolution X‐ray imaging. *Advanced Materials* 2023, 35(26): 2300136.

38. Shao W, Wang X, Zhang Z, Huang J, Han Z, Pi S*, et al.* Highly Efficient and Flexible Scintillation Screen Based on Manganese (II) Activated 2D Perovskite for Planar and Nonplanar High‐Resolution X‐Ray Imaging. *Advanced Optical Materials* 2022, 10(6): 2102282.

39. Su B, Han K, Xia Z. Mn^2+^-doped Cs_2_ZnBr_4_ scintillator for X-ray imaging. *Journal of Materials Chemistry C* 2023, 11(24): 8052-8061.
